# Supplementary material for: CEP signaling coordinates plant immunity with nitrogen status
Source: Nat Commun. 2024 Dec 16;15:10686. doi: 10.1038/s41467-024-55194-x (PMC11649690; doi:10.1038/s41467-024-55194-x)
Supplement: Supplementary file 1 — Supplementary information [file 41467_2024_55194_MOESM1_ESM.pdf]

CEP signaling coordinates plant immunity with nitrogen status

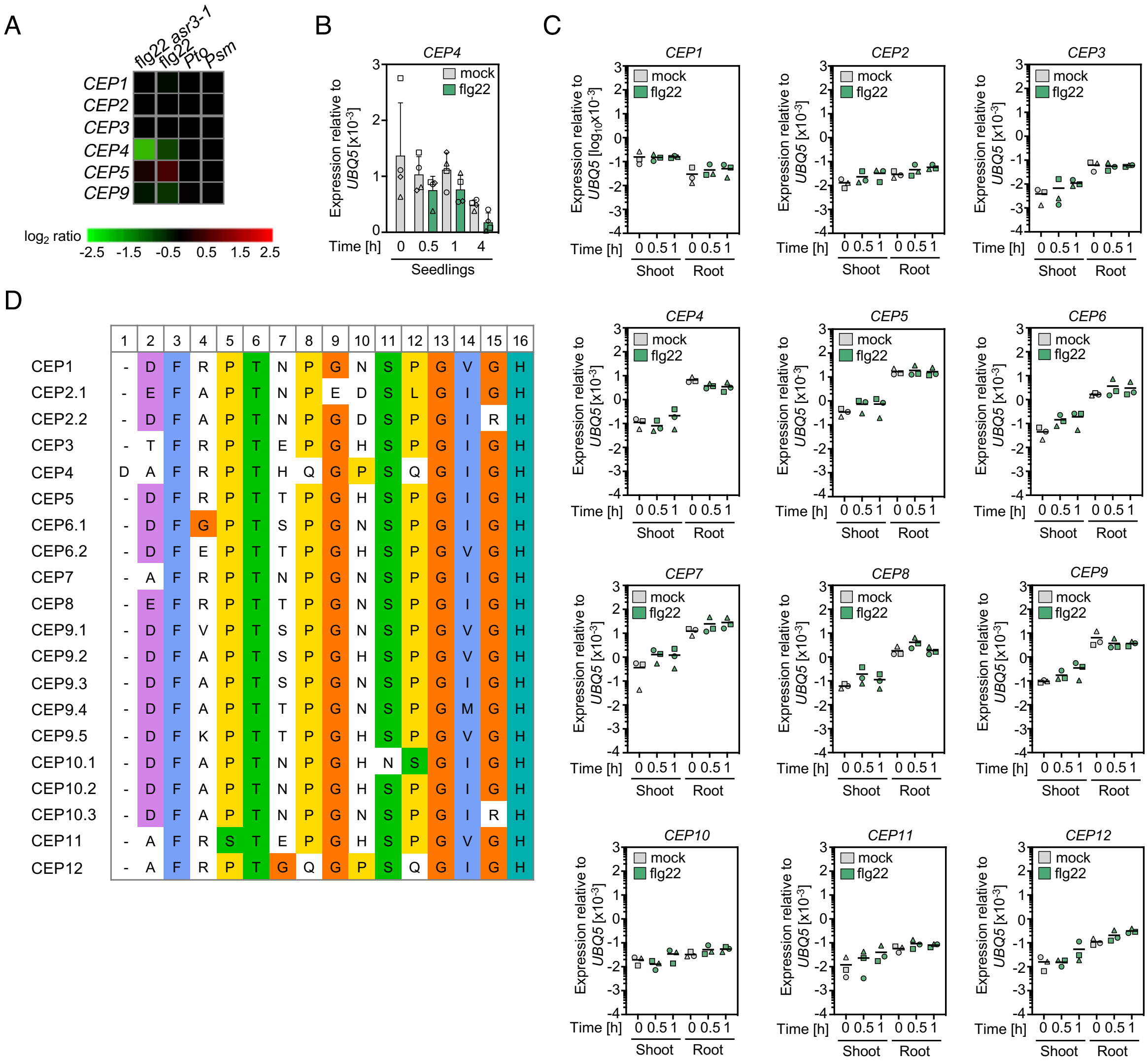

Supplementary Fig. 1: *CEP4* expression is differentially regulated after flg22 perception.
A) *CEP4* is downregulated after flg22 treatment in an *asr3* mutant background [33]. Gene expression data was extracted using the mRNA-seq data set AT_mRNASeq_ARABI_GL-1 from Genevestigator platform [103]. All *CEP* genes for which RNAseq data was available are depicted. **B)** *CEP4* expression in seedlings is weakly downregulated after flg22 treatment. Col-0 seedlings were treated with flg22 (100 nM) or mock (ddH_2O) for the indicated time, after which seedlings were harvested for RT-qPCR analysis of *CEP4* abundance. Housekeeping gene *UBQ5*; n = 4 from independent experiments, with mean ± SD. **C)** The majority of *CEPs* show higher expression in the roots. Col-0 seedlings were treated with flg22 (100 nM) or mock (ddH_2O) for the indicated time after which shoots and roots were harvested separately for RT-qPCR analysis. Housekeeping gene *UBQ5*; n = 3 from independent experiments, with mean ± SD. **D)** Alignment of group I CEP peptide domains from *Arabidopsis thaliana* marked with colours based on amino acid properties and conservation. The conserved proline (P) residues are indicated in yellow. Different symbols in **B** and **C** represent independent biological repeats.

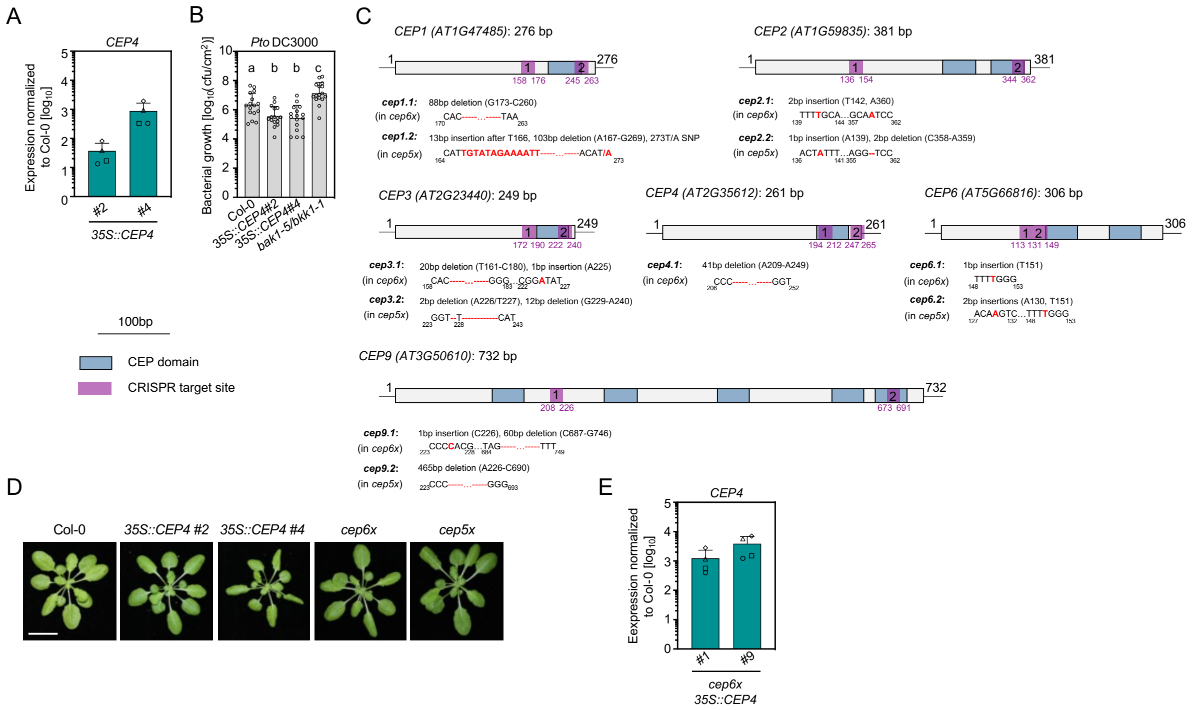

Supplementary Fig. 2: Characterization of *35S::CEP4*, CRISPR *cep* mutant alleles and *cep6x 35S::CEP4.*

A) *CEP4* transcript levels in two independent *CEP4* overexpression lines, shown as fold induction compared to Col-0. Housekeeping gene *UBQ5*; n = 4 from independent experiments, with mean ± SD. **B)** Cfu of *Pto* DC3000 (3 dpi) upon spray infection; n = 16 pooled from four independent experiments with mean ± SD (one-way ANOVA, Tukey post-hoc test; a-b/c $p<0.05$; b-c $p<0.0001$). **C)** Characterization of two independent *cep* mutant alleles. Schematic diagram of *CEP1*, *CEP2*, *CEP3*, *CEP4*, *CEP6* and *CEP9* gene structure and the CRISPR-Cas9-mediated mutation pattern detected by DNA sequencing. The locus number and the length of the coding sequence (CDS) are indicated above the scheme for each gene. CRISPR *cep6x* is mutated in *cep1/2/3/4/6/9* (alleles *cep1.1, cep2.1, cep3.1, cep4.1, cep6.1, cep9.1*), CRISPR *cep5x* is mutated in *cep1/2/3/6/9* with CEP4 wild-type (alleles *cep1.2, cep2.2, cep3.2, cep6.2, cep9.2*). The specific location and type of mutations for each gene are indicated in the schematics describing the mutants. The CEP domain is indicated in blue, and the two CRISPR target sites are indicated in purple; scale bar = 100 bp. **D)** Pictures of 5-week-old plants of the indicated genotypes grown on soil; scale bar = 2 cm. **E)** *CEP4* transcript levels in two independent lines of *cep6x 35S::CEP4* shown as fold induction compared to Col-0. Housekeeping gene *UBQ5*; n = 4 with mean ± SD. Different symbols in **A** and **E** represent independent biological repeats.

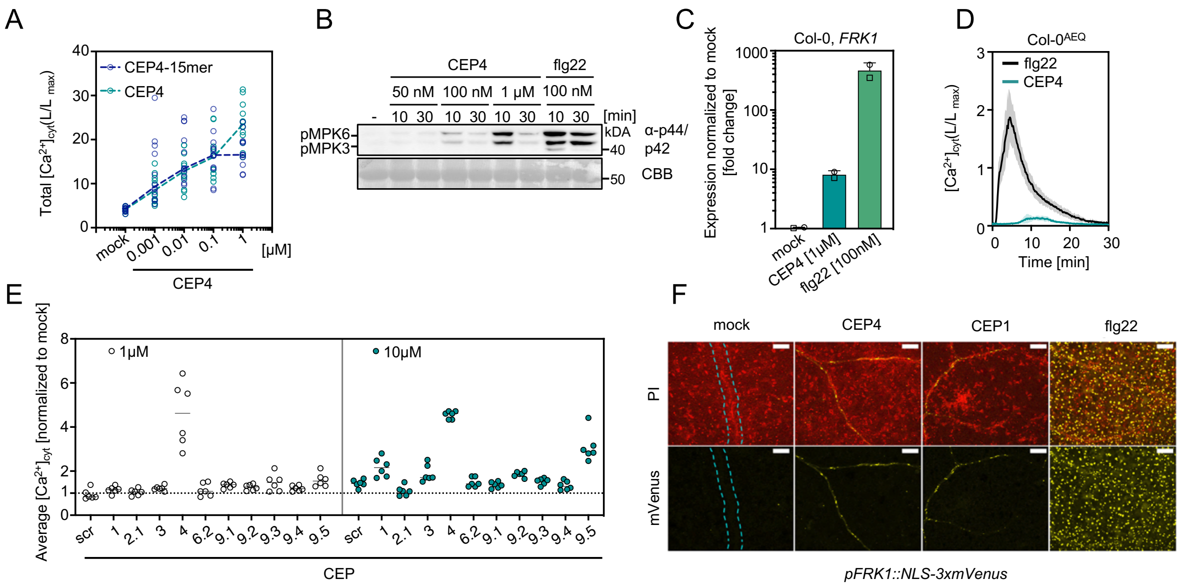

Supplementary Fig. 3: CEP4 is the strongest inducer of PTI responses on the whole tissue level.

A) Both *CEP4* variants trigger influx of calcium ions. Col-0^{AEQ} seedlings were treated with the indicated concentrations of CEP4 and CEP4-15mer. ($[Ca^{2+}]_{cyt}$) was measured for 30 min. Shown is total calcium influx with the line representing the mean; n = 12 pooled from two independent experiments. **B)** MAPK activation in Col-0 upon treatment with indicated concentrations of CEP4 or flg22. Western blots were probed with α-p44/42. Size marker is indicated. CBB = Coomassie brilliant blue. Similar results were obtained in three independent experiments. **C)** RT-qPCR of *FRK1* in seedlings upon mock (ddH_2O), CEP4 (1 µM) or flg22 (100 nM) treatment for 4 h, shown as fold induction compared to Col-0. Housekeeping gene *UBQ5*; n = 2 from independent experiments with mean ± SD. **D)** Kinetics of cytosolic calcium concentrations ($[Ca^{2+}]_{cyt}$) in Col-0^{AEQ} seedlings upon CEP4 (100 nM) and flg22 (100nM) treatment; n = 12, with mean ± SD. **E)** Some CEPs induce Ca^{2+} influx at higher concentrations. Col-0^{AEQ} seedlings were treated with the indicated concentrations of CEPs. ($[Ca^{2+}]_{cyt}$) was measured for 30 min. The average calcium influx triggered by individual CEPs is normalized to mock (ddH_2O). The line in the middle represents different experiments, and the dotted line allows for a comparison with mock. Similar results were obtained in two independent experiments. **F)** CEP4 and CEP1 induce *FRK1* promoter activity in the vasculature. Representative images of NLS-3xmVenus signal in *pFRK1::NLS-3xmVenus* lines following mock (ddH_2O), CEP1 (100 nM), CEP4 (100 nM) or flg22 (100 nM) treatment for 16 h. Maximum projection of Z-stack for mVenus merged with Z-stacked propidium iodide (PI) signal. Cyan dotted line indicates vasculature, scale bar = 100 µm. Similar results were obtained in three independent experiments.

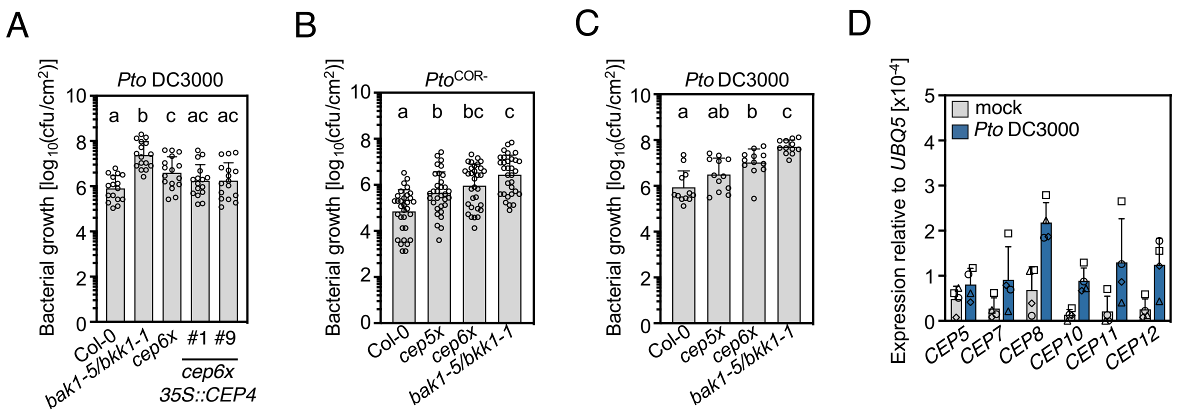

Supplementary Fig. 4: CEPs are important for resistance against *Pto*.

A) *35S::CEP4* partially rescues CRISPR *cep6x* susceptibility to *Pto* DC3000. Cfu of *Pto* DC3000 (3 dpi) upon spray infection; n = 16 pooled from four independent experiments with mean ± SD (one-way ANOVA, Tukey post-hoc test; a-b p<0.0001; a-c, b-c p<0.05; ac-b p≤0.0001). **B)** Loss of *CEPs* increases Arabidopsis susceptibility to Pto^{COR-}. Cfu of Pto^{COR-} (3 dpi) upon spray infection; n = 32 pooled from eight independent experiments with mean ± SD (Kruskal-Wallis, Dunn's post-hoc test; a-b p=0.0342; a-bc p=0.0008; a-c p <0.0001, b-c p=0.0166). **C)** Cfu of *Pto* DC3000 (3 dpi) upon spray infection; n = 12 pooled from three independent experiments with mean ± SD (one-way ANOVA, Tukey post-hoc test; a-b p=0.0004; a-c, ab-c p<0.0001). **D)** RT-qPCR analysis of *CEP* expression in mock (ddH_2O) and *Pto* DC3000-inoculated leaves 24 h post-treatment. Housekeeping gene *UBQ5*; n = 4 with mean ± SD and with different symbols showing independent biological repeats.

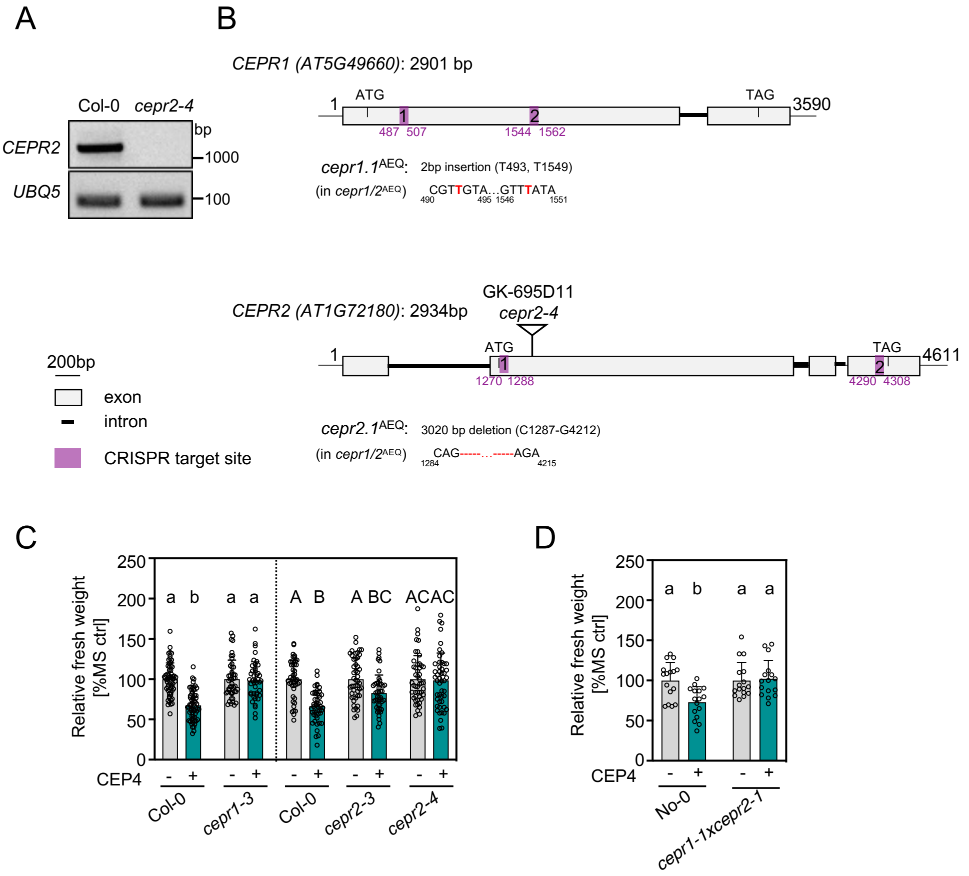

Supplementary Fig. 5: Characterization of *cepr2-4* and *cepr1/2*^{AEQ} mutants and CEP4-induced seedling growth inhibition.

A) Characterization of the *cepr2-4* mutant. The *CEPR2* transcript could not be detected in *cepr2-4* mutant in a semi-quantitative RT-PCR analysis. Housekeeping gene *UBQ5*. Size marker is indicated. **B)** Schematic diagram of the *CEPR1* and *CEPR2* genomic sequence, structure, and the CRISPR-Cas9-mediated mutation pattern detected by DNA sequencing. The locus number and the length of the coding sequence (CDS) are indicated above the scheme for each gene. The CRISPR *cepr1*/2^{AEQ} mutant was generated in Col-0^{AEQ} background. CRISPR *cepr1*^{AEQ} (in *cepr1/2*^{AEQ}) has two 1 bp insertions, which lead to a frameshift mutation and an early stop codon. CRISPR *cepr2*^{AEQ} (in *cepr1/2*^{AEQ}) has a 3020 bp deletion between the start and stop codon. The specific location and type of mutations for each gene are indicated in the schematics describing the mutants. The two CRISPR target sites are indicated in purple, exons are indicated in grey, and introns are shown as black lines. The T-DNA insertion site in *cepr2-4* is indicated with a black triangle; scale bar = 200 bp **C)** CEPR1 and CEPR2 are similarly required for CEP4-induced seedling growth inhibition. Relative fresh weight of five-day-old seedlings treated with CEP4 (1 µM) for seven days. The dotted line represents different experiments. Statistical analysis was performed separately for each group. The first group was pooled from five independent experiments with mean ± SD (Col-0 n = 60, *cepr1-3* mock n = 46, *cepr1-3* CEP4 n = 42; Kruskal-Wallis, Dunn's post-hoc test, a-b p<0.0001). The second group was pooled from four independent experiments with mean ± SD (Col-0 n = 46, *cepr2-3* mock n = 48, *cepr2-3* CEP4 n = 46, *cepr2-4* n = 48; Kruskal-Wallis, Dunn's post-hoc test, A-B, B-AC p<0.0001, A-BC p<0.05). **D)** CEP4-induced seedling growth inhibition is abolished in *cepr1-1xcepr2-1*. Relative fresh weight of five-day-old seedlings treated with CEP4 (1 µM) for seven days; n = 16 with mean ± SD (one-way ANOVA, Tukey post-hoc test, a-b p<0.01). Similar results were obtained in three independent biological repeats.

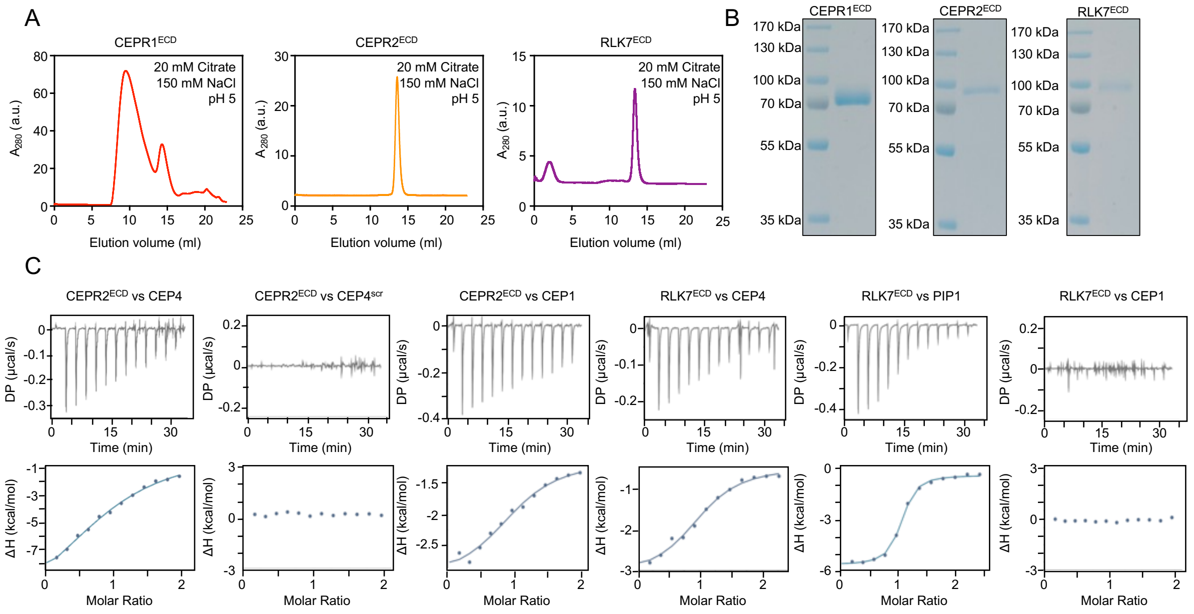

Supplementary Fig. 6: Quality control of recombinant CEPR1, CEPR2 and RLK7 ectodomains and supplemental ITC data.

A) Analytical size-exclusion chromatography (SEC) experiments show CEPR1^{ECD} aggregates in comparison to single peaks for CEPR2^{ECD} and RLK7^{ECD}. **B)** SDS-PAGE of the ECD peaks of the SEC analysis in **A**. **C)** ITC thermograms of the second technical repeat performed for each ectodomain and peptide analyzed in Fig. 2 (**D**-**F**) and Fig. 3 (**I**-**K**).

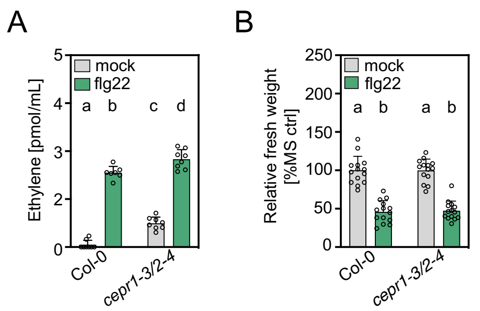

Supplementary Fig. 7: CEPR1 and CEPR2 are dispensable for flg22-induced ethylene accumulation and growth inhibition.

A) Basal and flg22-triggered ethylene production is higher in *cepr1-3/2-4* mutants. Ethylene concentration in leaf discs upon mock (ddH_2O) or CEP4 (1 µM) for 3.5 h; Col-0 flg22 n = 7, the rest n = 8 pooled from two independent experiments with mean ± SD (one-way ANOVA, Tukey post-hoc test, a-b/c/d, c-b/d p<0.0001, b-d p=0.0044). **B)** flg22-induced seedling growth inhibition is not affected in *cepr1-3/2-4*. Relative fresh weight of five-day-old seedlings treated with flg22 (100 nM) for seven days; n = 14 pooled from two independent experiments with mean ± SD (one-way ANOVA, Tukey post-hoc test, a-b p<0.0001).

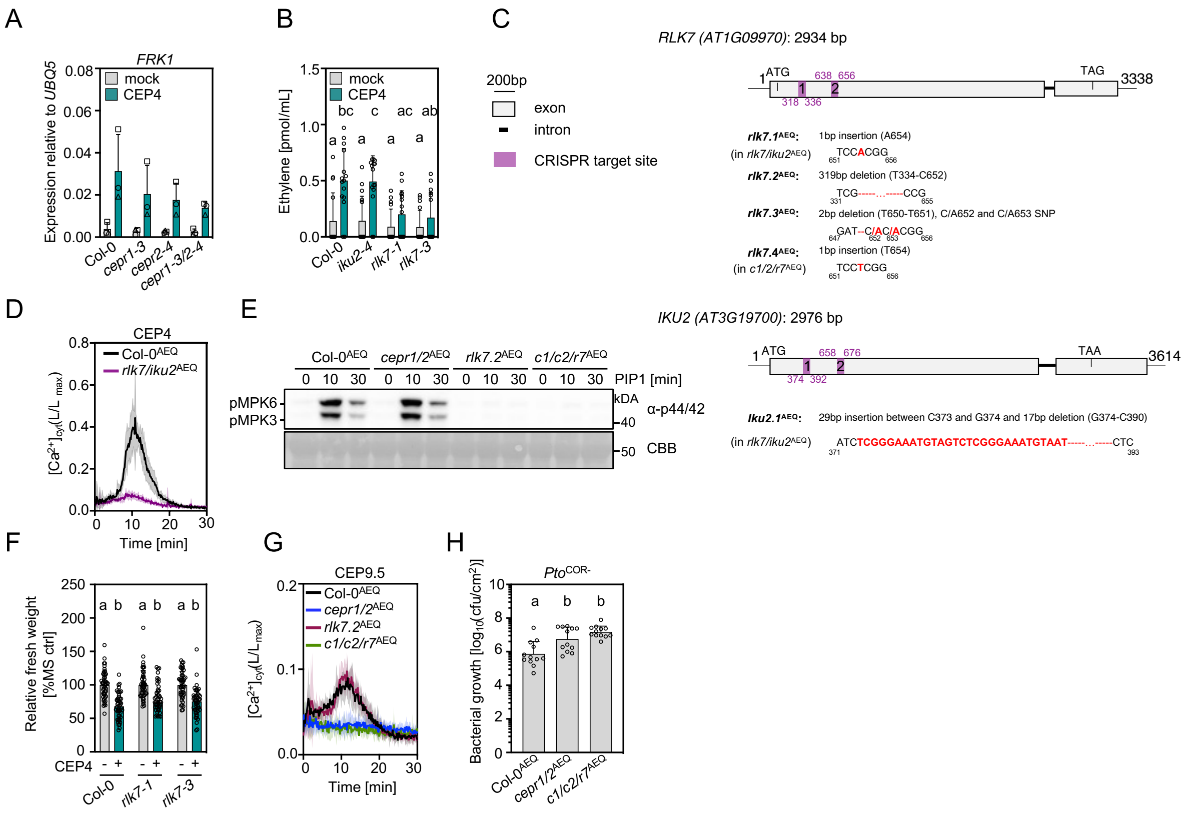

Supplementary Fig. 8: Identification of RLK7 as an additional CEP4 receptor.

A) RT-qPCR of *FRK1* in seedlings upon mock (ddH$_2$O) or CEP4 (1 μM) treatment for 4 h. Housekeeping gene *UBQ5*; n = 3 with different symbols showing independent biological repeats. **B)** CEP4-triggered ethylene production is reduced in *rlk7* mutants. Ethylene concentration in leaf discs upon mock (ddH$_2$O) or CEP4 (1 μM) treatment for 3.5 h; n = 15 pooled from three independent experiments with mean ± SD (Kruskal-Wallis, Dunn's post-hoc test, a-bc p=0.0147, a-c p=0.0062, ab-c p=0.0265). **C)** Schematic diagram of the *RLK7* and *IKU2* genomic sequence, structure, and the CRISPR-Cas9-mediated mutation pattern detected by DNA sequencing. The locus number and the length of the coding sequence (CDS) are indicated above the scheme for each gene. There are four *rlk7* alleles generated in Col-0^{AEQ} background: *rlk7.1*^{AEQ} in *rlk7/iku2*^{AEQ} double mutant, two single *rlk7*^{AEQ} mutants: *rlk7.2*^{AEQ} and *rlk7.3*^{AEQ}, and *rlk7.4*^{AEQ} in CRISPR *cepr1/2/rlk7*^{AEQ}. CRISPR *iku2* (in *rlk7/iku2*^{AEQ}) has a 29 bp insertion, followed by a 17 bp deletion. Mutations in both genes lead to a frameshift mutation and an early stop codon. The specific location and type of mutations for each gene are indicated in the schematics describing the mutants. The two CRISPR target sites are indicated in purple, exons are indicated in grey, and introns are shown as black lines. **D)** $[Ca^{2+}]_{cyt}$ kinetics in seedlings upon CEP4 treatment (1 μM); n = 6 with mean ± SD. Similar results were obtained in three independent experiments. **E)** MAPK activation upon PIP1 (1 μM) treatment. Western blots were probed with α-p44/42. Size marker is indicated. CBB = Coomassie brilliant blue. Identical results were obtained in two independent experiments. **F)** Relative fresh weight of five-day-old seedlings treated with CEP4 (1 μM) for seven days; n = 48 pooled from four independent experiments with mean ± SD (Kruskal-Wallis, Dunn's post-hoc test, a-b p<0.0005). **G)** $[Ca^{2+}]_{cyt}$ kinetics in seedlings upon CEP9.5 (10 μM) treatment; n = 6 with mean ± SD. Same results were obtained in three independent experiments. **H)** cfu of *Pto*$^{COR-}$ (3 dpi) upon spray infection; n = 12 pooled from three independent experiments with mean ± SD (Welch's ANOVA, Dunnett's T3 post-hoc test; a-b, p<0.05).

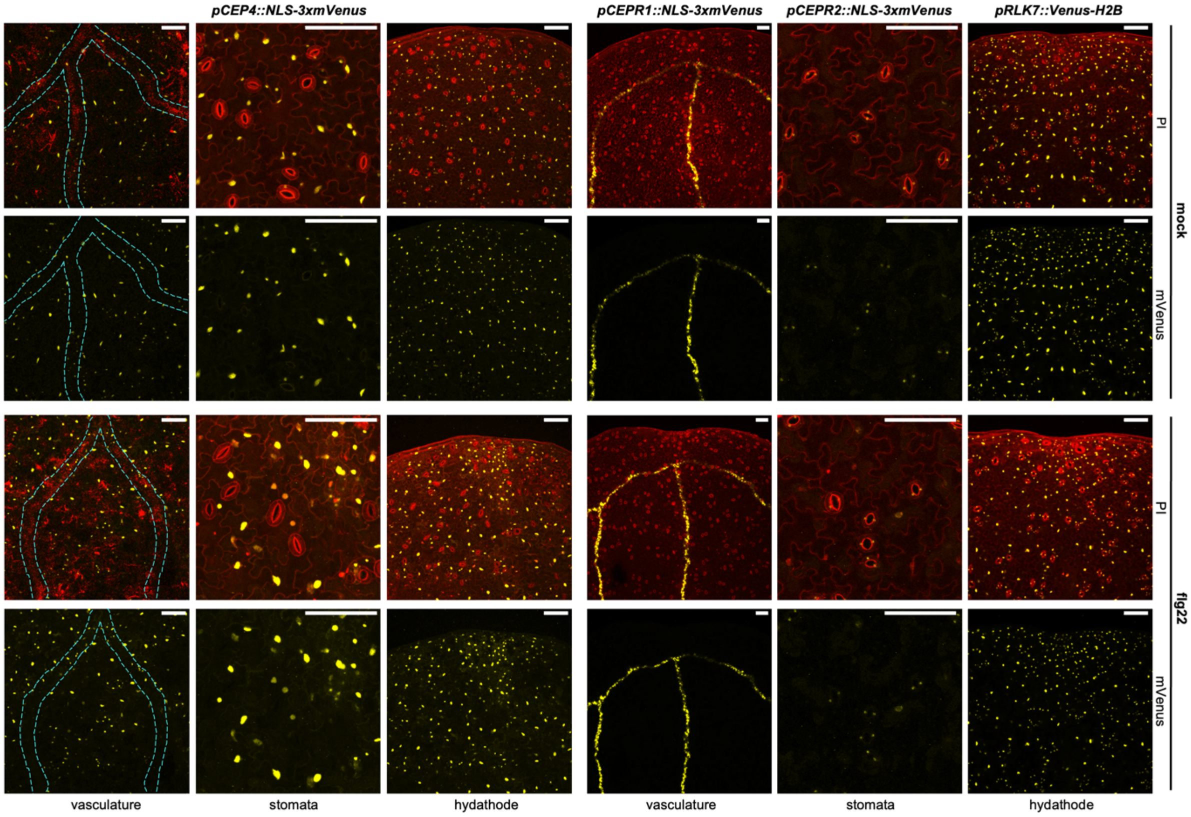

Supplementary Fig. 9: Tissue-specific promoter activity of CEP4 and cognate CEP receptors does not change after flg22 perception.

Representative images of nuclear mVenus signal in the indicated reporter lines upon mock (ddH_2O) and flg22 (100 nM) treatment for 16 h, shown as the maximum projection of Z-stacks for mVenus merged with Z-stacked propidium iodide (PI) signal. Cyan dotted lines indicate vasculature, scale bar = 100 µm. For comparison purposes, cotyledons of 12-day-old seedlings of the same genotype were imaged using identical laser intensities and interval/number of slices for Z-stack projection in all images captured in one experiment. The experiment was repeated at least two times in independent biological repeats with similar results.

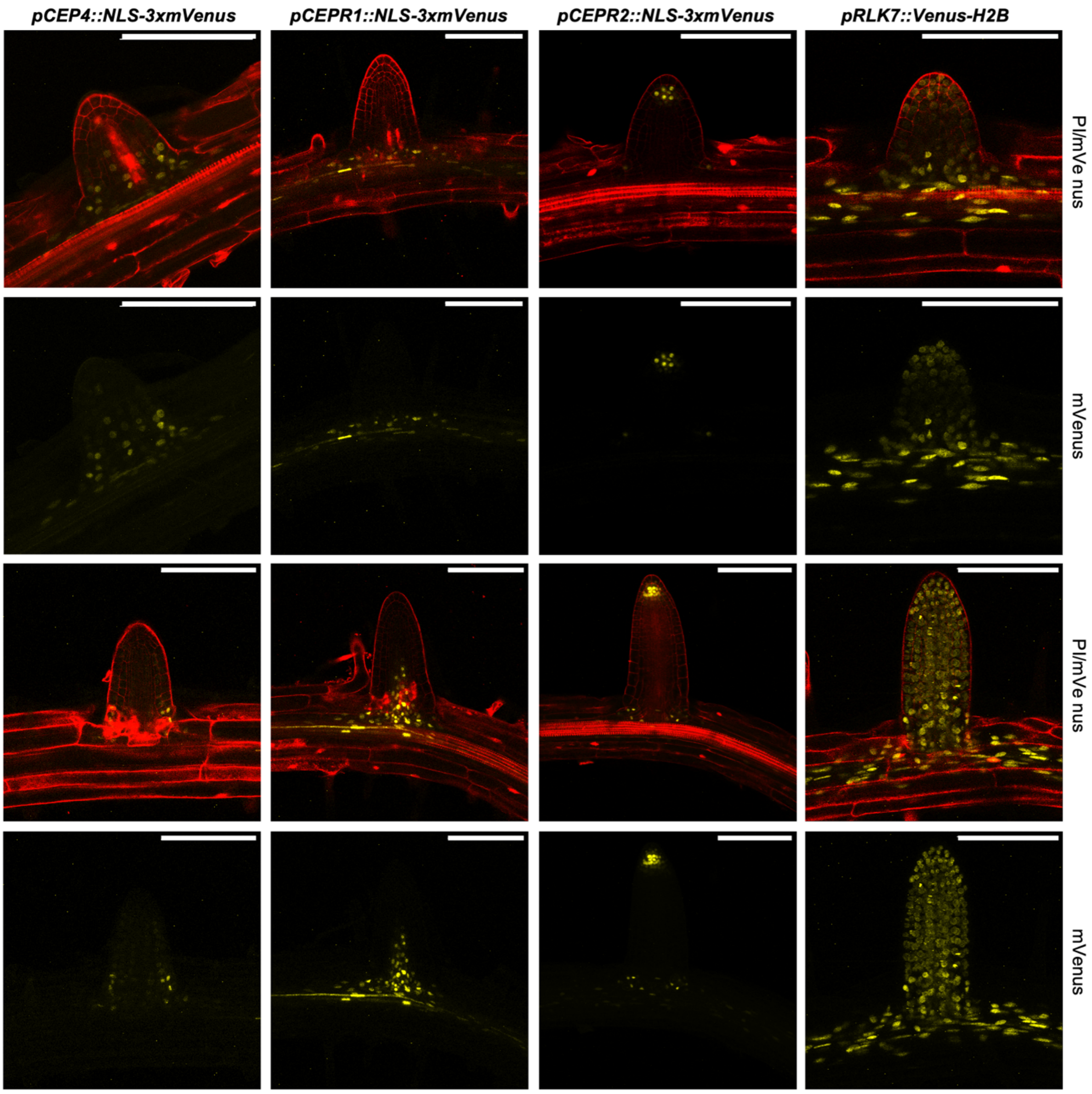

Supplementary Fig. 10: Promoter activity of CEP4 and cognate CEP receptors might overlap at the base of young lateral roots.

Representative images of nuclear mVenus signal in lateral roots of the indicated reporter lines, shown as a maximum projection of Z-stack for mVenus merged with a single layer of propidium iodide (PI) signal; scale bar = 100 µm. The laser power used for excitation varied across different reporter lines depending on the activity level of the tested promoters. Similarly, the Z-stack step size varied between reporter lines due to a different tissue-specific expression pattern of each promoter and was adjusted to capture all the cell layers where the signal was active. The experiment was repeated three times in independent biological repeats with similar results.

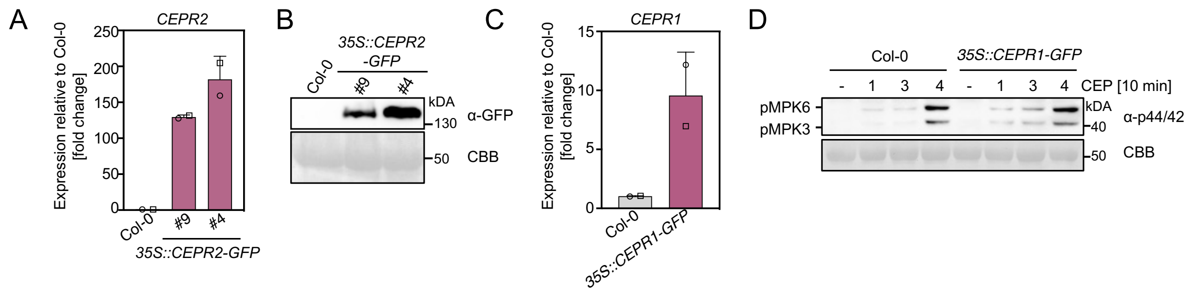

Supplementary Fig. 11: Characterization of *35S::CEPR1-GFP* and *35S::CEPR2-GFP* overexpression lines.

A) *CEPR2* transcript levels in two independent *CEPR2-GFP* overexpression lines. *CEPR2* expression was normalized to *UBQ5* and is shown as fold induction compared to Col-0; n = 2 from independent experiments with mean ± SD. **B)** CEPR2-GFP protein levels in wild-type Col-0 and *CEPR2* overexpression lines. Western blots were probed with α-GFP. Size marker is indicated. CBB = Coomassie brilliant blue. **C)** *CEPR1* transcript levels in the *CEPR1-GFP* overexpression line. *CEPR1* expression was normalized to *UBQ5* and is shown as fold induction compared to Col-0; n = 2 from independent experiments with mean ± SD. **D)** MAPK activation in Col-0 and *35S::CEPR1-GFP* line upon CEP1 (1), CEP3 (3) and CEP4 (4) 1 µM treatment. Western blots were probed with α-p44/42. Size marker is indicated. CBB = Coomassie brilliant blue. Different symbols in **A** and **C** represent independent experiments. All experiments were performed twice in independent biological repeats with similar results.

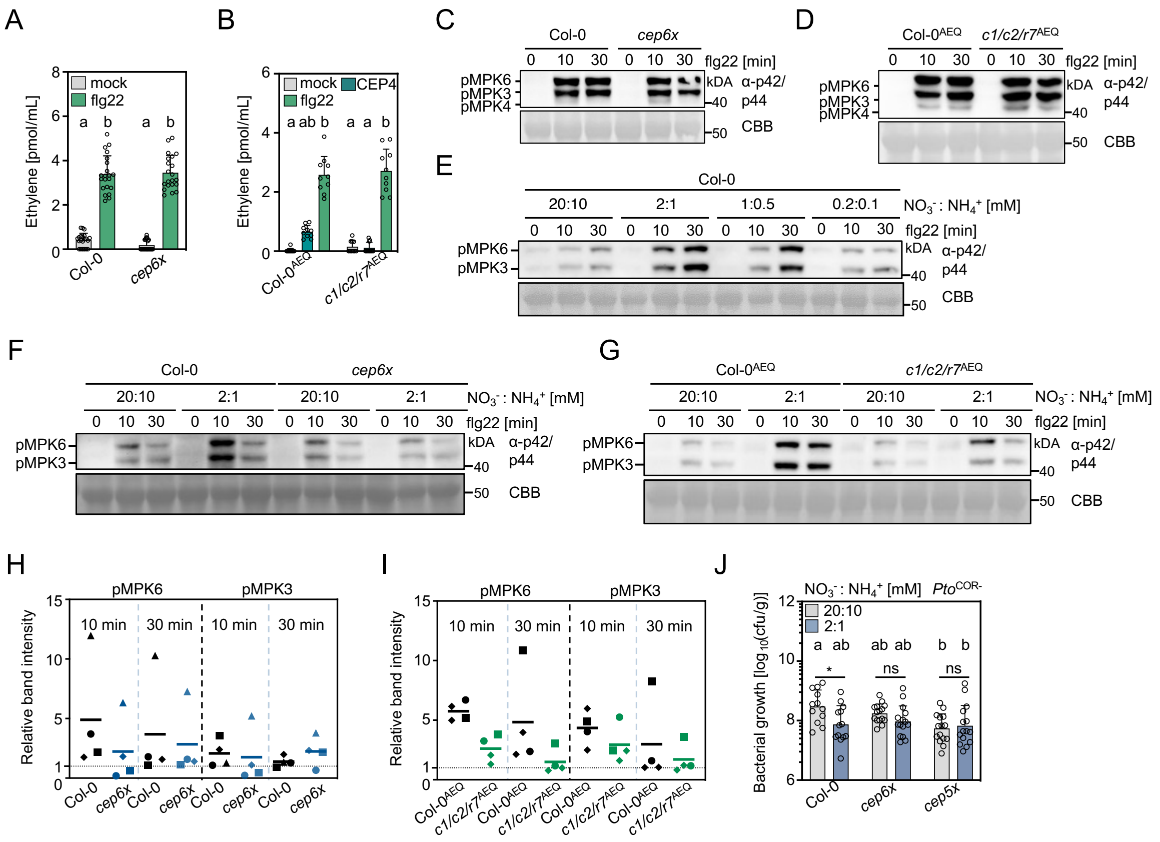

Supplementary Fig. 12: Further characterization of CRISPR *cep* and CRISPR *cepr1/2/rlk7*^{AEQ} mutants.

A) Flg22-triggered ethylene production is not impaired in *cep6x*. Ethylene concentration in leaf discs upon mock (ddH_2O) or flg22 (500 nM) treatment for 3.5 h; n = 21 pooled from four independent experiments with mean ± SD (Kruskal-Wallis, Dunn's post-hoc test, a-b p<0.0001). **B)** flg22-triggered ethylene production is not impaired in *cepr1/2/rlk7*^{AEQ}. Ethylene concentration in leaf discs upon mock (ddH_2O), CEP4 (1 µM) or flg22 (500 nM) treatment for 3.5 h; n = 10 pooled from two independent experiments with mean ± SD (Kruskal-Wallis, Dunn's post-hoc test, a-b p<0.0001). *cep6x* **(C)** and *cepr1/2/rlk7*^{AEQ} **(D)** are not affected in flg22-triggered MAPK activation under standard conditions. MAPK activation upon flg22 (100 nM) treatment. **E)** N availability modulates flg22-triggered MAPK activation. MAPK activation upon flg22 (100 nM) treatment after 24 h transfer of seedlings to medium containing indicated concentrations of N. **F)** and **G)** CEP signaling is important for N-dependent modulation of FLS2 response. MAPK activation upon flg22 (100 nM) treatment after 24 h transfer of seedlings to medium containing indicated concentrations of N. **H)** and **I)** Quantification of pMPK6/pMPK3 band intensities normalized to the CBB band and relative to flg22-treated standard N (20 mM NO_3^- : 10 mM NH_4^+) of the respective genotype (set as 1 using ImageJ software); n = 4, with mean. Different symbols represent independent experiments. **J)** cfu of *Pto*$^{COR-}$ (3 dpi) upon flood inoculation. Col-0 (n = 12), *cep6x* and *cep5x* (n = 15) for standard N and Col-0 (n = 13), *cep6x* and *cep5x* (n = 15) for reduced N, pooled from three independent experiments with mean ± SD (general comparison between genotypes: one-way ANOVA, Tukey post-hoc test, a-b p<0.05; comparison within the same genotype: two-tailed Student's t-test, * p=0.0188). Western blots were probed with α-p44/42. Size marker is indicated. CBB = Coomassie brilliant blue. The experiments in **C**-**G** were performed at least three times in independent biological repeats with similar results.

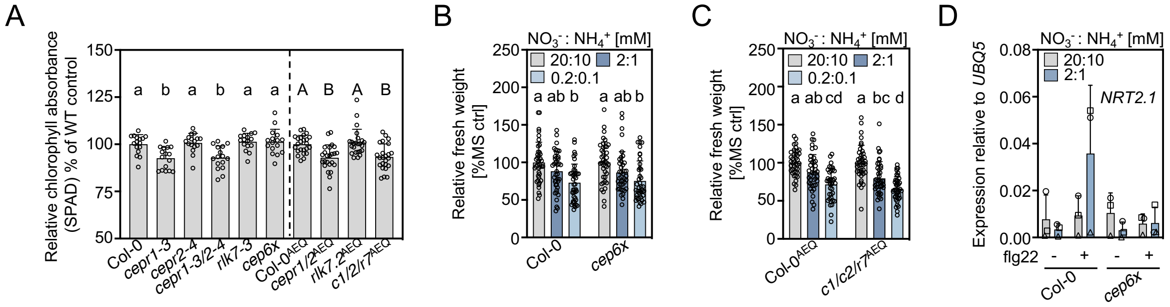

Supplementary Fig. 13: *cep6x* is not impaired in chlorophyll content and shows wild-type growth response to different medium N concentrations.

A) Relative chlorophyll absorbance, correlating to chlorophyll content shown in SPAD (Soil Plant Analysis Development) units. The dotted line indicates different experiments; n = 16 and 24 pooled from two and three independent experiments, respectively, with mean ± SD (one-way ANOVA, Tukey post-hoc test, a-b p<0.01, A-B p<0.005). **B)** The *cep6x* mutant shows wild-type growth response to different medium N concentrations. Relative fresh weight of five-day-old seedlings grown in medium containing indicated concentrations of N for seven days; n = 40 pooled from five independent experiments with mean ± SD (Kruskal-Wallis, Dunn's post-hoc test, a-b p≤0.0005). **C**) The *cepr1/2/rlk7*^{AEQ} shows a slightly stronger growth response to different medium N concentrations. Relative fresh weight of five-day-old seedlings grown in medium containing indicated concentrations of N for seven days; n = 39 (the lowest N Col-0^{AEQ}), the rest n = 40 pooled from five independent experiments with mean ± SD (one-way ANOVA, Tukey post-hoc test, a-cd, a-d, ab-d p<0.0001, bc-d p=0.0303). **D)** RT-qPCR of *NRT2.1* upon flg22 (100 nM) treatment of seedlings for 4 h after 24 h transfer to medium containing indicated concentrations of N. Housekeeping gene *UBQ5*; n = 3 with mean ± SD. Different symbols represent independent experiments.

Supplementary Table 1: Peptides used in this study.

Peptide name	Sequence
CEP1	DFRHypTNPGNSHypGVGH
CEP2.1	DFAHypTNPGDSHypGIRH
CEP3	TFRHypTEPGHSHypGIGH
CEP4^{scr}	TGQHypDHQRHypFAHIGGS
CEP4^{15mer}	AFRHypTHQGHypSQGIGH
CEP4^{16mer}	DAFRHypTHQGHypSQGIGH
CEP6.2	DFEPTTHypGHSHypGVGH
CEP9.1	DFVHypTSHypGNSHypGVGH
CEP9.2	DFAHypTSHypGHSHypGVGH
CEP9.3	DFAHypTSHypGNSHypGIGH
CEP9.4	DFAHypTTHypGNSHypGMGH
CEP9.5	DFKPTTHypGHSHypGVGH
PIP1	RLASGHypSPRGRGH
flg22	Ac-QRLSTGSRINSAKDDAAGLQIA

Hyp (in red) indicates hydroxylated proline residues.

Supplementary Table 2: CRISPR-Cas9 target sites.

Target site	Sequence	Purpose
CEP1.1	TGGTCACATATACACGGCG	Generation of CRISPR *cep* mutants
CEP1.2	CAGGCGTTGGACACTCTAA	
CEP2.1	GTCCCGGTATCAGGCATCC	
CEP2.2	ATGTATGCCCTGCAAAAGT	
CEP3.1	TACGGAATGTCCAATACCG	
CEP3.2	TATCAACCCCGTGGCTCGG	
CEP4.1	CATTCCGACCAACCCACCA	
CEP4.2	TATTTTAAGGAGCACCTGG	
CEP6.1	ATGATCATCATTTCACAGT	
CEP6.2	TCGGGTACACTGATGATTT	
CEP9.1	CAAGATGACTTCAAGCCCA	
CEP9.2	ACACCAGGACATAGCCCCG	
CEPR1.1	AGAGAGACGAAGTACACGG	Generation of *cepr1/2*^{AEQ} mutant
CEPR1.2	AGCTCATGAGGTATAACAC	
CEPR2.1	GACCTCCTACGTGGCAGCG	
CEPR2.2	CTCAGACCGAGTATGAGAG	
RLK7.1	AGATTGATCTCTCTCGTCG	Generation of *rlk7*^{AEQ} and *rlk7/iku2*^{AEQ} mutants
RLK7.2	GCGACGGCTGATTTCCCGG	
IKU2.1	GCTTGAGATACTTAGACCT	Generation of *iku2*^{AEQ} mutant
IKU2.2	CTTGTTCGTCTACAAAACT	

Supplementary Table 3: Primers used in this study.

Primers used for cloning		
**Primer name**	**Sequence (5'→3')**	**Purpose**
pCEPR1-BsaI-F	GCGCGCGGTCTCTCGAGTCAGTTTTTTTGGATTCAGATTTG	*pro*CEPR1 for pCEPR1::NLS-3xmVenus-tNOS
pCEPR1-BsaI-R	GCGCGCGGTCTCTCATTTTTCAGAGAAAGATCAAAAGTAACC	
pCEPR2-BsaI-F	GCGCGCGGTCTCTCGAGTTTTGTATACTCTTAAAAAAACTATG	*pro*CEPR2 for pCEPR2::NLS-3xmVenus-tNOS
pCEPR2-BsaI-R	GCGCGCGGTCTCTCATTTTATAGTGATTCCCAAGGG	
pCEP4-BpiI-F	TTGAAGACTTCGAGAATATAAACAAATATAGGTAAC	*pro*CEP4 for pCEP4::NLS-3xmVenus-t35S
pCEP4-BpiI-R	TTGAAGACTTCATTCACTTGACAACTTTCTG	
pRLK7-AttB1	GGGGACAAGTTTGTACAAAAAAGCAGGCTTAGAGCTAAGACATATAACT	*pro*RLK7 for pRLK7-H2B-mVenus
pRLK7-AttB2	GGGGACCACTTTGTACAAGAAAGCTGGGTAGTCGGAGAGAAGAAGTGG	
CEPR1-AttB-F	GGGGACAAGTTTGTACAAAAAAGCAGGCTTTTATGCGTCTCAAAAATTTCCC	35S::CEPR1-GFP
CEPR1-AttB-R	GGGGACCACTTTGTACAAGAAAGCTGGGTCGAGTCTTGTTTGCGTGAG	
CEPR2-AttB-F	GGGGACAAGTTTGTACAAAAAAGCAGGCTATGTCGAGAAGACCAGACC	35S::CEPR2-GFP
CEPR2-AttB-R	GGGGACCACTTTGTACAAGAAAGCTGGGTCTACTGTAATCTTTCCAGTTGTGTC	
**Primers used for RT-qPCR**		
**Primer name**	**Sequence (5'→3')**	**Purpose**
qUBQ5-F	ACTCCTTCCTCAAACGCTGA	*UBQ5*
qUBQ5-R	CCAAGCCGAAGAAGATCAAG	
qFRK1-F	TGCAGCGCAAGGACTAGAG	*FRK1*
qFRK1-R	ATCTTCGCTTGGAGCTTCTC	
qPR1-F	CGGAGCTACGCAGAACAACT	*PR1*
qPR1-R	CAGACAAGTCACCGCTACCC	
qNRT2.1-F	AACAAGGGCTAACGTGGATG	*NRT2.1*
qNRT2.1-R	CTGCTTCTCCTGCTCATTCC	
qCEP1-F	AATGCTAAAGGGGTGTTTGG	*CEP1*
qCEP1-R	ACAAACCCACGACAAAGACA	
qCEP2-F	TGGTGACCATTTTGACCATC	*CEP2*
qCEP2-R	CCGACCATCTTTTTCGACTT	
qCEP3-F	GACCTACGGAACCTGGTCAT	*CEP3*
qCEP3-R	AAAAAGTCACCAGGCCAATC	
qCEP4-F	AGAATACAAAAGCAGCTCGAC	*CEP4*
qCEP4-R	TCCAATACCTTGACTAGGACC	
qCEP5-F	CCATGGACGAACCCTAAAAG	*CEP5*
qCEP5-R	TGCCATCATCGTCTTGCTAT	
qCEP6-F	GTCGGGTAATCTAGACCTTCCTC	*CEP6*
qCEP6-R	CGGTTTTTCGCAACTGTCTCG	

qCEP7-F	AGAATCTCACATCGTTGAAGGTCG	*CEP7*
qCEP7-R	GTTGCCCGCAGCGAATTTC	
qCEP8-F	CAGAGGCAAGACACTTGAGGAC	*CEP8*
qCEP8-R	TCTTCACAGAGCCACCACCG	
qCEP9-F	AGCTGGATTTACAGATGATTTCG	*CEP9*
qCEP9-R	TGTCCCACACCAGGACTGT	
qCEP10-F	TTGTGGTGACCAGTTTAACCA	*CEP10*
qCEP10-R	TGCTCATGACGGTCCATCTT	
qCEP11-F	CGTTCAAGCCATCACCATCATC	*CEP11*
qCEP11-R	CTGTGACCTGGTTCAGTAGACC	
qCEP12-F	ACCAGTTAGAGTGTTTGGTCCG	*CEP12*
qCEP12-R	GGGTCGGAATGCAGTTTGTTC	
qCEPR1-F	ATGCGTACTCGTCCAAAGCA	*CEPR1*
qCEPR1-R	CTCCCCGAAACACGAATCCA	
qCEPR2-F	CTTCAAAAACCGCCTCGACG	*CEPR2*
qCEPR2-R	CCGATGACTTCGCCGGATAA	
**Primers used for genotyping and sequencing**		
**Primer name**	**Sequence (5'→3')**	**Purpose**
GG pUC18 Seq-F	GTATCACGAGGCCCTTTCGT	Inserts in GoldenGate pUC18
GG pUC18 Seq-R	TAATGAATCGGCCAACGC	
CEP1-CRISP-s1	CCTTGTTTTGTTTTACATATTCTT	CRISPR CEP1
CEP1-CRISP-s2	TCTGAATGAAAATACATGTAATTTTC	
CEP2-CRISP-s1	CATTCAATACTTCTCATATACAAAAC	CRISPR CEP2
CEP2-CRISP-s2	TCACCAGTTTTCTTTAGTCTTC	
CEP3-CRISP-s1	CATTTTCGCCTTCGACTA	CRISPR CEP3
CEP3-CRISP-s2	CAATCATATTTTCAACATGTAATCAC	
CEP4-CRISP-s1	GACATACGCTTTTAAAGGAAG	CRISPR CEP4
CEP4-CRISP-s2	GAAGAACTGTGTGCGATAAAG	
CEP6-CRISP-s1	CCTCTCTCGTATACACTAGAAAC	CRISPR CEP6
CEP6-CRISP-s2	TTAATTTAGCAAAAGCATTGAAG	
CEP9-CRISP-s1	ATATATACAAACTCCTAAACCTTGC	CRISPR CEP9
CEP9-CRISP-s2	CATATCGTAAACCAATGTATTAGA	
CEPR1-CRISP-s1	CGTAAGATGTGACGGTCAA	CRISPR CEPR1
CEPR1-CRISP-s2	GGTTGCCTTGCAGTACAA	
CEPR2-CRISP-s1	GAATTCCAGTGCATCGACC	CRISPR CEPR2
CEPR2-CRISP-s2	CTAAAACGTGGTAGGTGAATCAT	
RLK7-CRISP-s1	TCAAACCACCGTCACCACTT	CRISPR RLK7
RLK7-CRISP-s2	TCCAAATTCCGAAGCTCCGT	
IKU2-CRISP-s1	GCATGTGAATTCGCCGGAAT	CRISPR IKU2
IKU2-CRISP-s2	CCGCAGCTCTGATAAATCACC	
pCEPR1-s1	CTAGTCACCTGCAGCTCG	pCEPR1
pCEPR1-s2	GTGTAGTTCACGTCGGATG	
pCEPR1-s3	CAAACGCTTCACTTATGTAATG	
pCEPR1-s4	GCATCTATCTGATTCTGATCGTG	
pCEPR1-s5	CATCCGACGTGAACTACAC	
pCEPR2-s1	ACGACAATCTGAGCTCCAC	pCEPR2
pCEPR2-s2	TGGGATTCGGAGCATAAGG	
pCEPR2-s3	TCCATCTAGAAACATTAACCGA	
pCEPR2-s4	CCTAGAACGTGGTCCAAG	
pCEPR2-s5	CTGCACTGTAATGAGTACC	

pCEPR2-s6	CGGTAATAGATAGTGAAATGG	
cepr1-3-LP	TAAATCAAGAATCCACTTCCATGC	*cepr1-3*
cepr1-3-RP	CCTAATGGTAACTTATGGGACGCT	
cepr2-4-LP	GGTGAGTTCGTTATCGCTGAG	*cepr2-4*
cepr2-4-RP	AATGTTGAATCGACCGTTGAG	
GABI_o8474	ATAATAACGCTGCGGACATCTACATTTT	T-DNA primer for GABI-kat
cepr2-3-LP	TCACAACTCTGTAACGCAACG	*cepr2-3*
cepr2-3-RP	AACTCGGAGTTTTGAAGGAGC	
rlk7-1-LP	CCGCCTCTCTCTCTCTCTCTC	*rlk7-1*
rlk7-1-RP	AAGCAGAGCTTTCATCTTCCC	
rlk7-3-LP	ACGATTTGATCGTCGTGCTAC	*rlk7-3*
rlk7-3-RP	TTACAACAACTCCTTGACCGG	
iku2-4-LP	TCTTTAAGAACCGCAGCTCTG	*iku2-4*
iku2-4-RP	GTTGTTTCGCCTACAATGACC	
LBb1.3	ATTTTGCCGATTTCGGAAC	T-DNA LB primer for *rlk7-1, rlk7-3, iku2-4*
LBb1	GCGTGGACCGCTTGCTGCAACT	T-DNA LB primer for *cepr2-3*

Supplementary references

103. Hruz, T. et al, Genevestigator V3: A Reference Expression Database for the Meta-Analysis of Transcriptomes. *Adv. Bioinforma*. **2008**, 420747 (2008).
