# Supplementary material for: CEP signaling coordinates plant immunity with nitrogen status
Source: Nat Commun. 2024 Dec 16;15:10686. doi: 10.1038/s41467-024-55194-x (PMC11649690; doi:10.1038/s41467-024-55194-x)
Supplement: Supplementary file 3 — Reporting Summary [file 41467_2024_55194_MOESM3_ESM.pdf]

nature portfolio

Corresponding author(s): Martin Stegmann

Last updated by author(s): 30/10/2024

Reporting Summary

Nature Portfolio wishes to improve the reproducibility of the work that we publish. This form provides structure for consistency and transparency in reporting. For further information on Nature Portfolio policies, see our Editorial Policies and the Editorial Policy Checklist.

Statistics

For all statistical analyses, confirm that the following items are present in the figure legend, table legend, main text, or Methods section.

n/a	Confirmed	
☐	☒	The exact sample size (*n*) for each experimental group/condition, given as a discrete number and unit of measurement
☐	☒	A statement on whether measurements were taken from distinct samples or whether the same sample was measured repeatedly
☐	☒	The statistical test(s) used AND whether they are one- or two-sided<br>*Only common tests should be described solely by name; describe more complex techniques in the Methods section.*
☒	☐	A description of all covariates tested
☐	☒	A description of any assumptions or corrections, such as tests of normality and adjustment for multiple comparisons
☐	☒	A full description of the statistical parameters including central tendency (e.g. means) or other basic estimates (e.g. regression coefficient) AND variation (e.g. standard deviation) or associated estimates of uncertainty (e.g. confidence intervals)
☐	☒	For null hypothesis testing, the test statistic (e.g. *F*, *t*, *r*) with confidence intervals, effect sizes, degrees of freedom and *P* value noted<br>*Give P values as exact values whenever suitable.*
☒	☐	For Bayesian analysis, information on the choice of priors and Markov chain Monte Carlo settings
☒	☐	For hierarchical and complex designs, identification of the appropriate level for tests and full reporting of outcomes
☒	☐	Estimates of effect sizes (e.g. Cohen's *d*, Pearson's *r*), indicating how they were calculated

Our web collection on statistics for biologists contains articles on many of the points above.

Software and code

Policy information about availability of computer code

Data collection

qPCR data: AriaMx Real-Time PCR system (Agilent, USA),
Ethylene data: Varian 3300 gas chromatograph
Calcium data: Luminoskan Ascent plate reader (Thermo, USA)
Microscopy: Leica TCS SP5 (Leica, Germany)
Peptide binding: MicroCal PEAQ-ITC (Malvern Instruments, UK)
Chlorophyll content: SPAD-502 (Minolta, Tokyo, Japan)
Western blot: Fusion SL Vilber Lourmat (Vilber, France)

Data analysis

qPCR: AriaMx software Version 2.0
Calcium data: Luminoskan Ascent 2.1 software
Data processing: Microsoft Excel 2019
Statistical analysis and figure generation: GraphPad Prism Version 10.2.3
ITC analysis: MicroCal PEAQ-ITC Analysis Software
Western blot development and quantification: Fusion Software, ImageJ (version 1.53t)

For manuscripts utilizing custom algorithms or software that are central to the research but not yet described in published literature, software must be made available to editors and reviewers. We strongly encourage code deposition in a community repository (e.g. GitHub). See the Nature Portfolio guidelines for submitting code & software for further information.

Data

Policy information about availability of data

All manuscripts must include a data availability statement. This statement should provide the following information, where applicable:

- Accession codes, unique identifiers, or web links for publicly available datasets
- A description of any restrictions on data availability
- For clinical datasets or third party data, please ensure that the statement adheres to our policy

All data generated in this study are available in the main text or the Supplementary Information. All newly generated mutant lines are available upon request to M.S. Source data are provided with this paper

Human research participants

Policy information about studies involving human research participants and Sex and Gender in Research.

Reporting on sex and gender	n/a
Population characteristics	n/a
Recruitment	n/a
Ethics oversight	n/a

Note that full information on the approval of the study protocol must also be provided in the manuscript.

Field-specific reporting

Please select the one below that is the best fit for your research. If you are not sure, read the appropriate sections before making your selection.

☒ Life sciences ☐ Behavioural & social sciences ☐ Ecological, evolutionary & environmental sciences

For a reference copy of the document with all sections, see nature.com/documents/nr-reporting-summary-flat.pdf

Life sciences study design

All studies must disclose on these points even when the disclosure is negative.

Sample size	No sample size calculation was performed. Sample size was determined according to standards in the field and literature. Given the sample size, adequate statistical analysis was performed.
Data exclusions	No data were excluded from analysis.
Replication	All data was replicated with the number of replicates provided in the figure legends.
Randomization	Plants were grown in random locations in the growth chamber. Plant samples were collected randomly.
Blinding	Blinding was not required for the assays used in this study.

Reporting for specific materials, systems and methods

We require information from authors about some types of materials, experimental systems and methods used in many studies. Here, indicate whether each material, system or method listed is relevant to your study. If you are not sure if a list item applies to your research, read the appropriate section before selecting a response.

Materials & experimental systems

n/a	Involved in the study	
☐	☒	Antibodies
☐	☒	Eukaryotic cell lines
☒	☐	Palaeontology and archaeology
☒	☐	Animals and other organisms
☒	☐	Clinical data
☒	☐	Dual use research of concern

Methods

n/a	Involved in the study	
☒	☐	ChIP-seq
☒	☐	Flow cytometry
☒	☐	MRI-based neuroimaging

Antibodies

Antibodies used	α-p44/42 (Cell Signaling, USA)<br>α-rabbit IgG-HRP sc2357 (Santa Cruz, USA)<br>α-GFP 3H9 (Chromotek, Germany)<br>α-rat-HRP A9542 (Sigma Aldrich, USA)
Validation	Commercial antibodies were used as recommended by manufacturers.

Eukaryotic cell lines

Policy information about cell lines and Sex and Gender in Research

Cell line source(s)	BTI-TNAO38
Authentication	none of the cells used were authenticated
Mycoplasma contamination	no mycoplasma contamination was detected
Commonly misidentified lines<br>(See ICLAC register)	not applicable
