# Supplementary material for: CEP signaling coordinates plant immunity with nitrogen status
Source: Nat Commun. 2024 Dec 16;15:10686. doi: 10.1038/s41467-024-55194-x (PMC11649690; doi:10.1038/s41467-024-55194-x)
Supplement: Supplementary file 4 — Source Data [file 41467_2024_55194_MOESM4_ESM.zip › !Source_data/Source_data_blots_gels.pptx]

## Slide 1
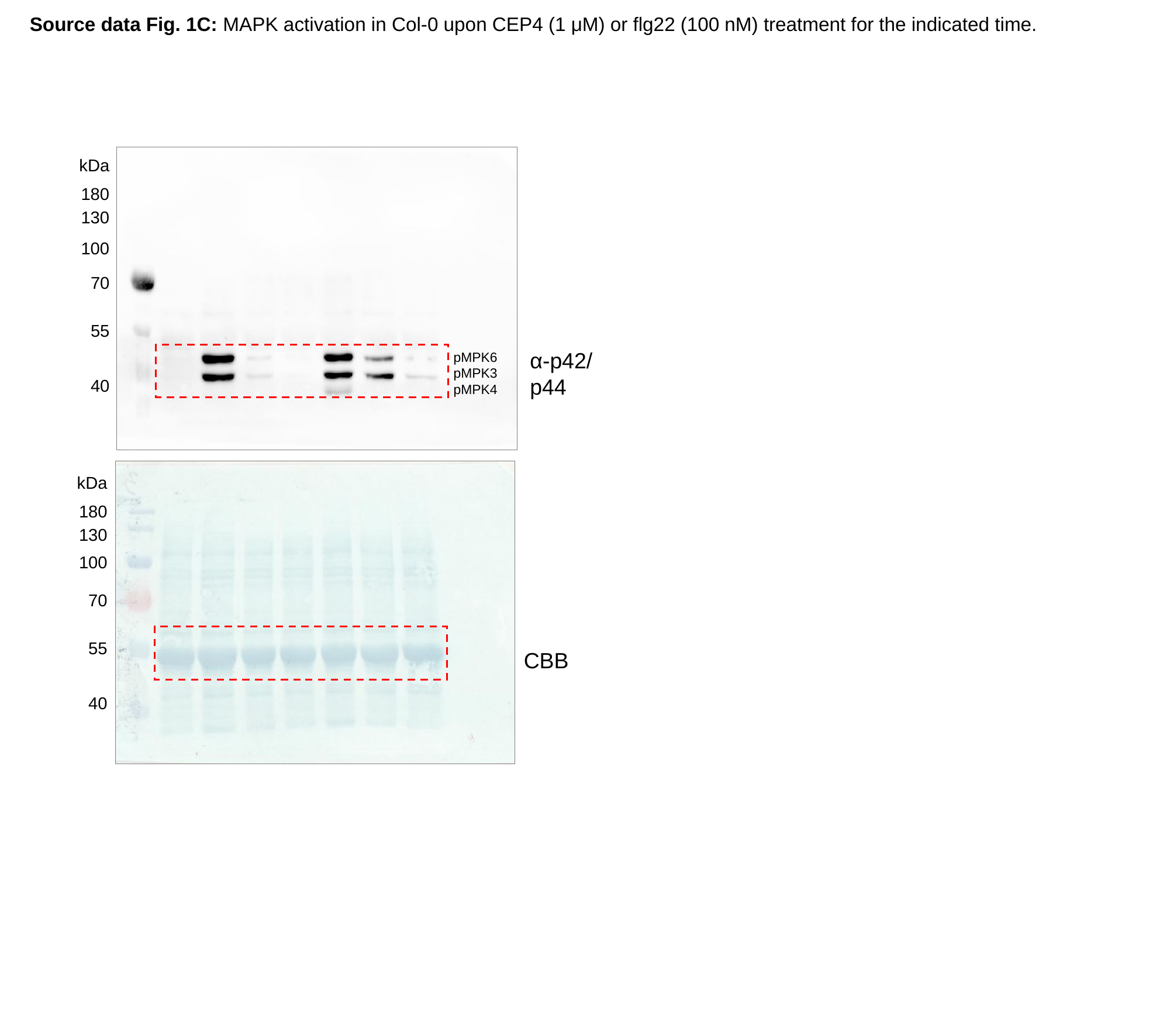

Source data Fig. 1C: MAPK activation in Col-0 upon CEP4 (1 μM) or flg22 (100 nM) treatment for the indicated time.
kDa
180
130
100
70
55
α-p42/
p44
pMPK6
pMPK3
pMPK4
40
kDa
180
130
100
70
55
CBB
40

## Slide 2
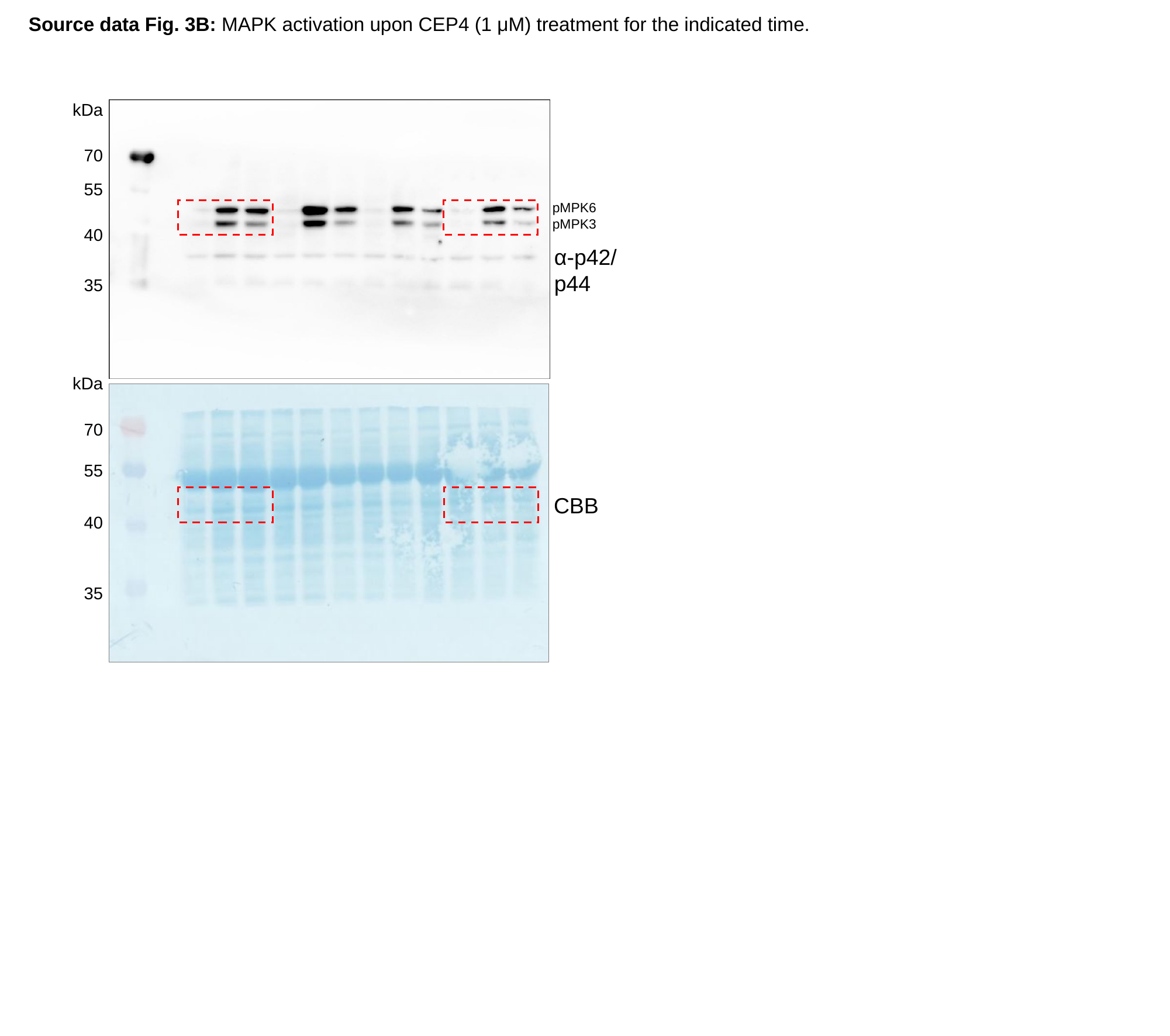

Source data Fig. 3B: MAPK activation upon CEP4 (1 μM) treatment for the indicated time.
kDa
70
55
pMPK6
pMPK3
40
α-p42/
p44
35
kDa
70
55
CBB
40
35

## Slide 3
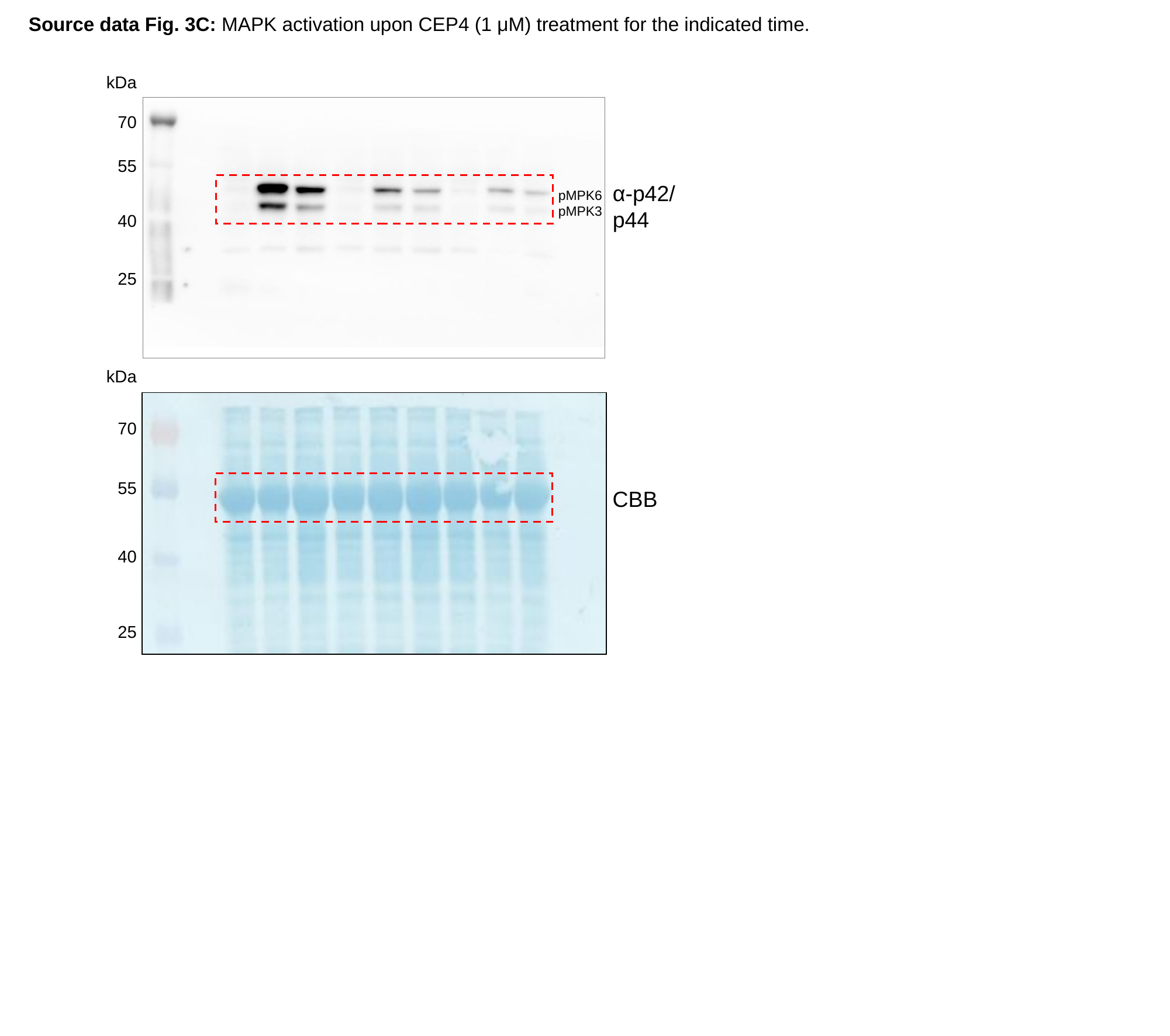

Source data Fig. 3C: MAPK activation upon CEP4 (1 μM) treatment for the indicated time.
kDa
70
55
α-p42/
p44
pMPK6
pMPK3
40
25
kDa
70
55
CBB
40
25

## Slide 4
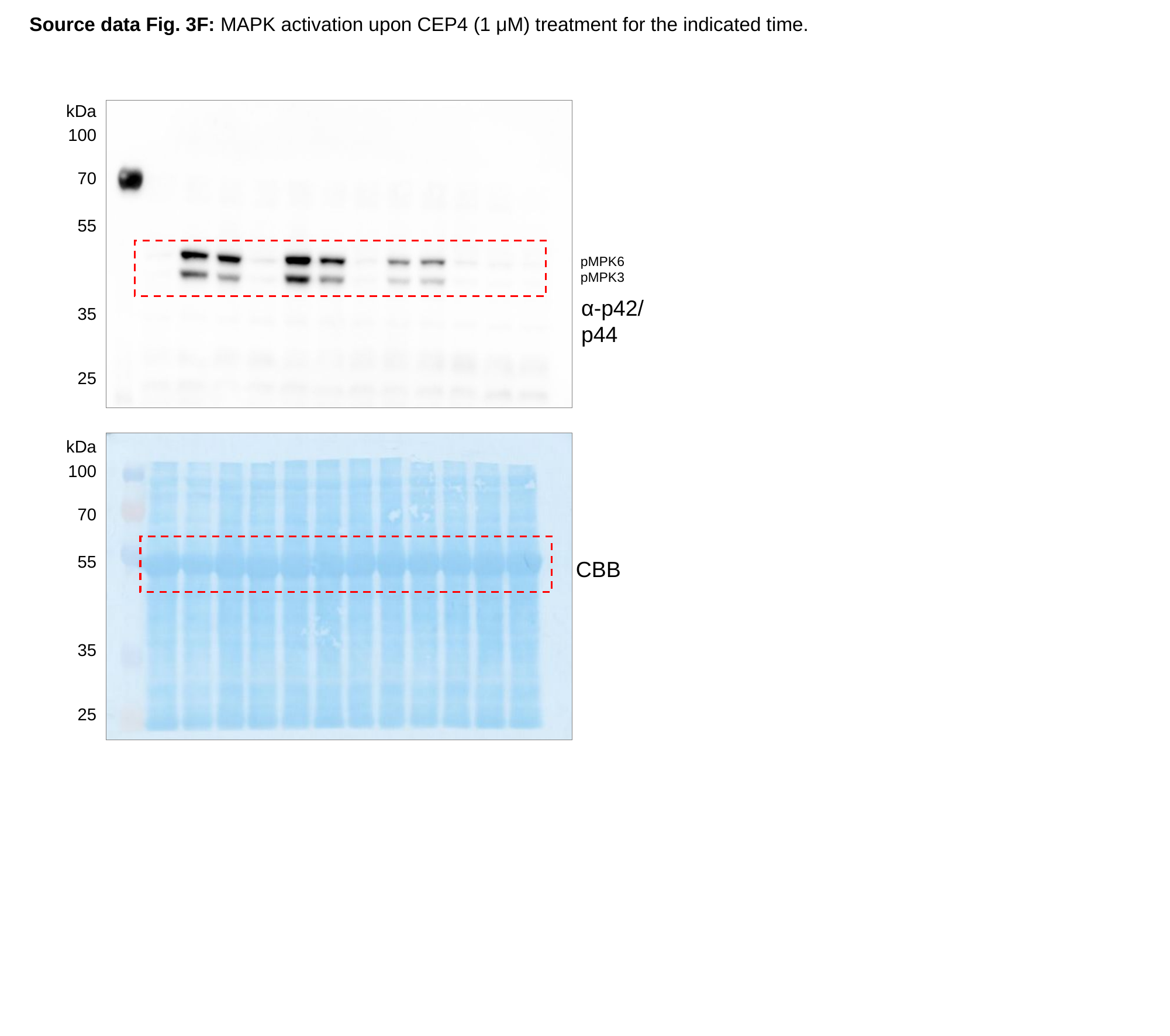

Source data Fig. 3F: MAPK activation upon CEP4 (1 μM) treatment for the indicated time.
kDa
100
70
55
pMPK6
pMPK3
α-p42/
p44
35
25
kDa
100
70
55
CBB
35
25

## Slide 5
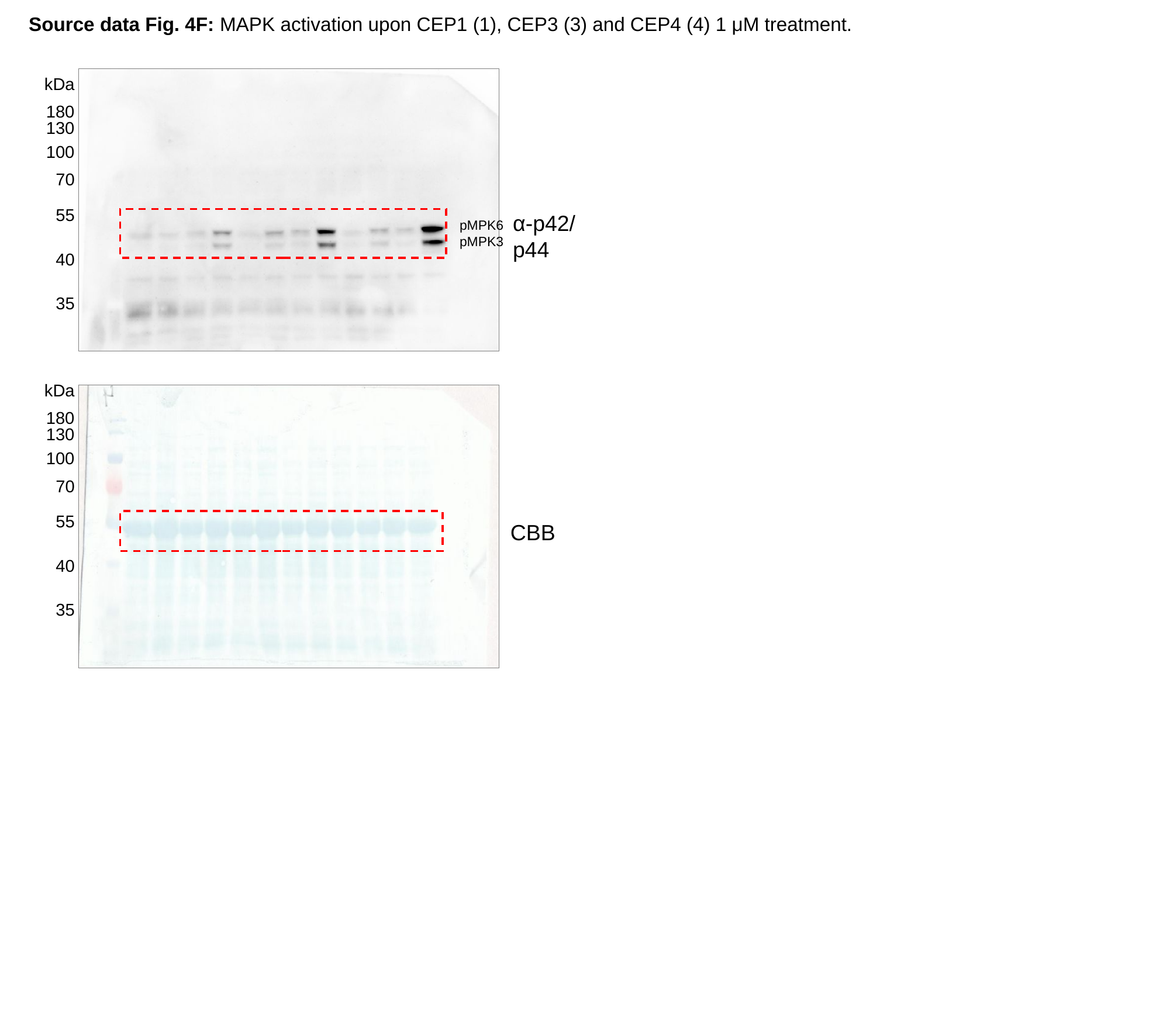

Source data Fig. 4F: MAPK activation upon CEP1 (1), CEP3 (3) and CEP4 (4) 1 μM treatment.
kDa
180
130
100
70
55
α-p42/
p44
pMPK6
pMPK3
40
35
kDa
180
130
100
70
55
CBB
40
35

## Slide 6
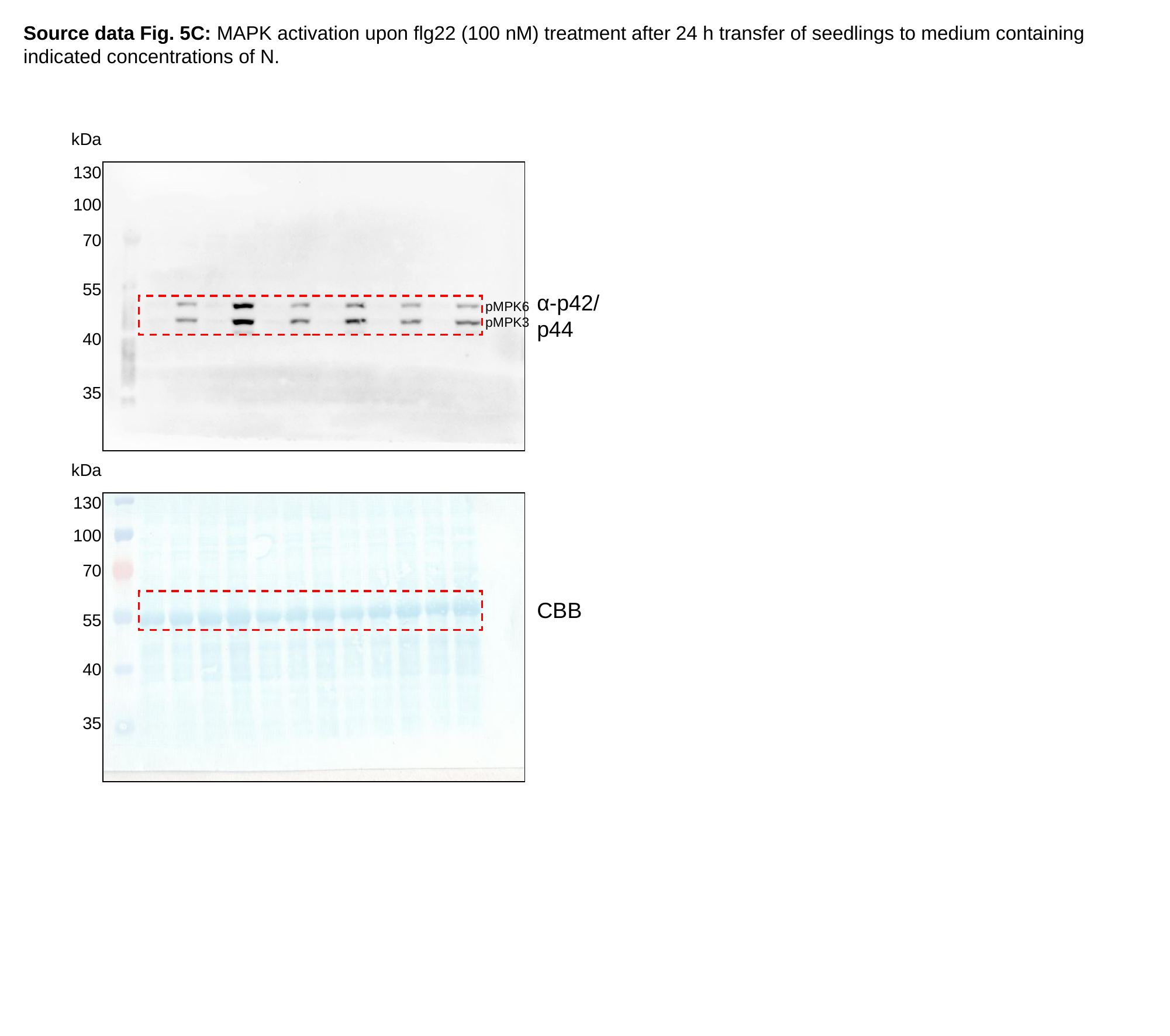

Source data Fig. 5C: MAPK activation upon flg22 (100 nM) treatment after 24 h transfer of seedlings to medium containing indicated concentrations of N.
kDa
130
100
70
55
α-p42/
p44
pMPK6
pMPK3
40
35
kDa
130
100
70
CBB
55
40
35

## Slide 7
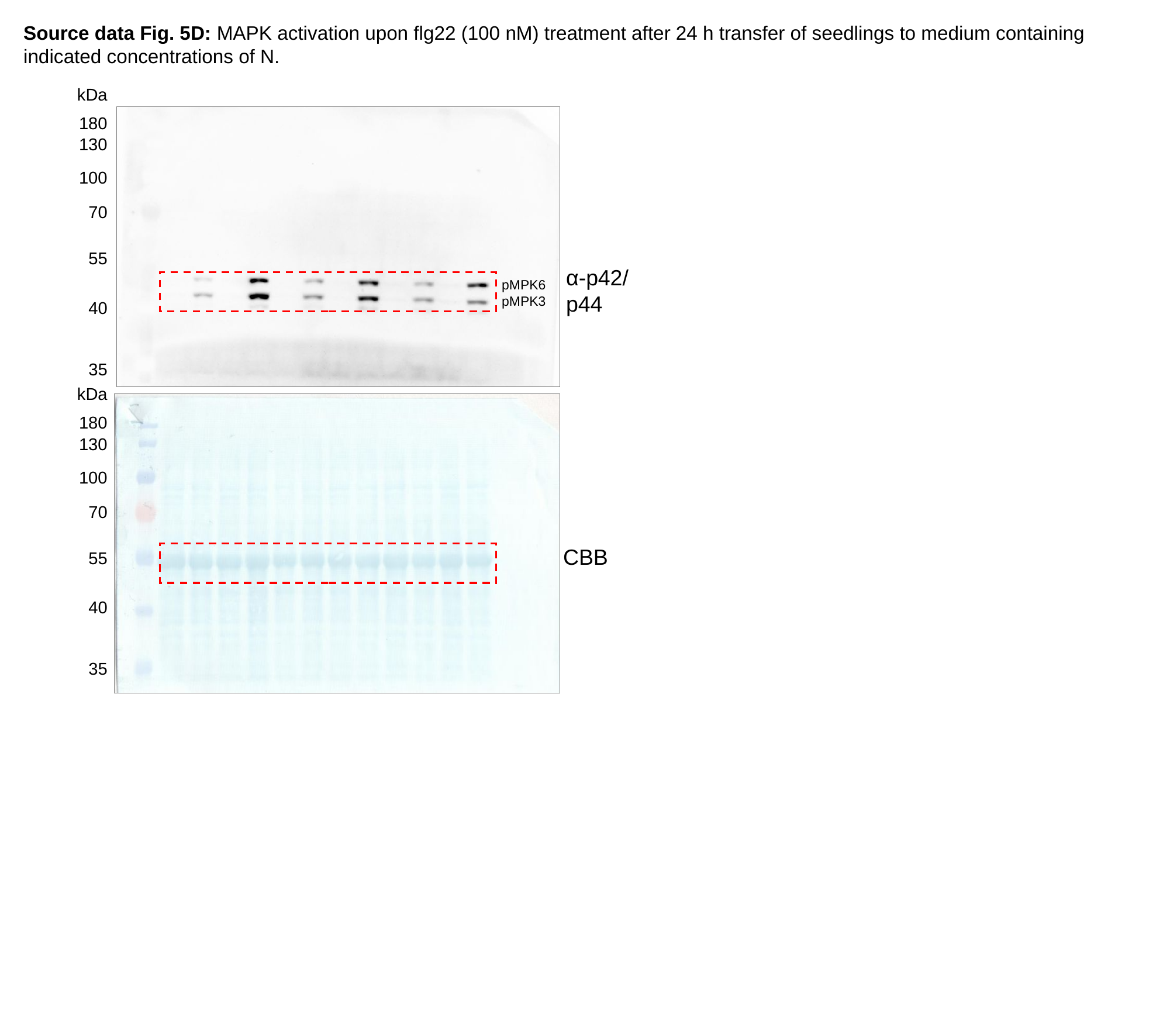

Source data Fig. 5D: MAPK activation upon flg22 (100 nM) treatment after 24 h transfer of seedlings to medium containing indicated concentrations of N.
kDa
180
130
100
70
55
α-p42/
p44
pMPK6
pMPK3
40
35
kDa
180
130
100
20:1
70
CBB
55
40
35

## Slide 8
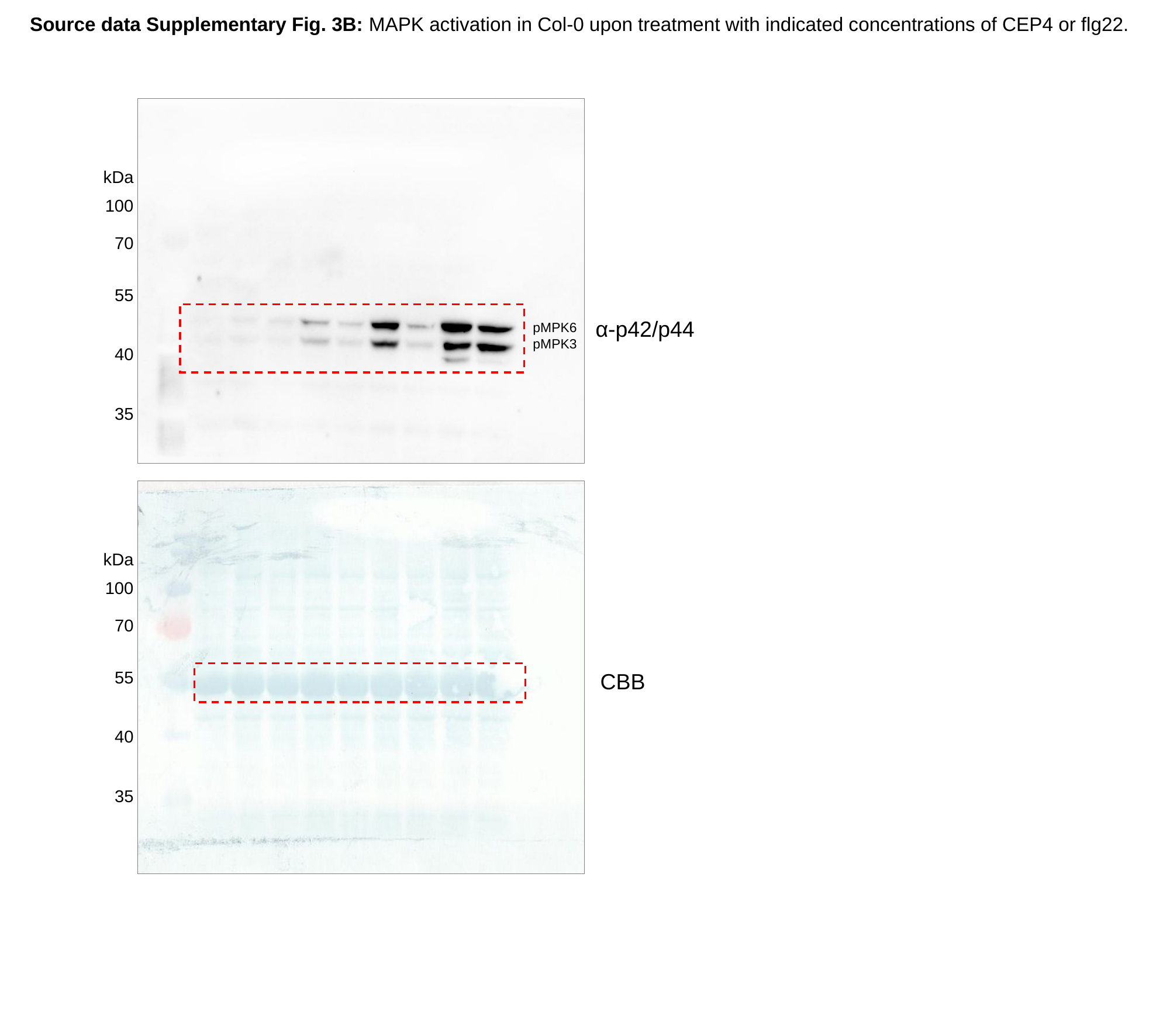

Source data Supplementary Fig. 3B: MAPK activation in Col-0 upon treatment with indicated concentrations of CEP4 or flg22.
kDa
100
70
55
α-p42/p44
pMPK6
pMPK3
40
35
kDa
100
70
55
CBB
40
35

## Slide 9
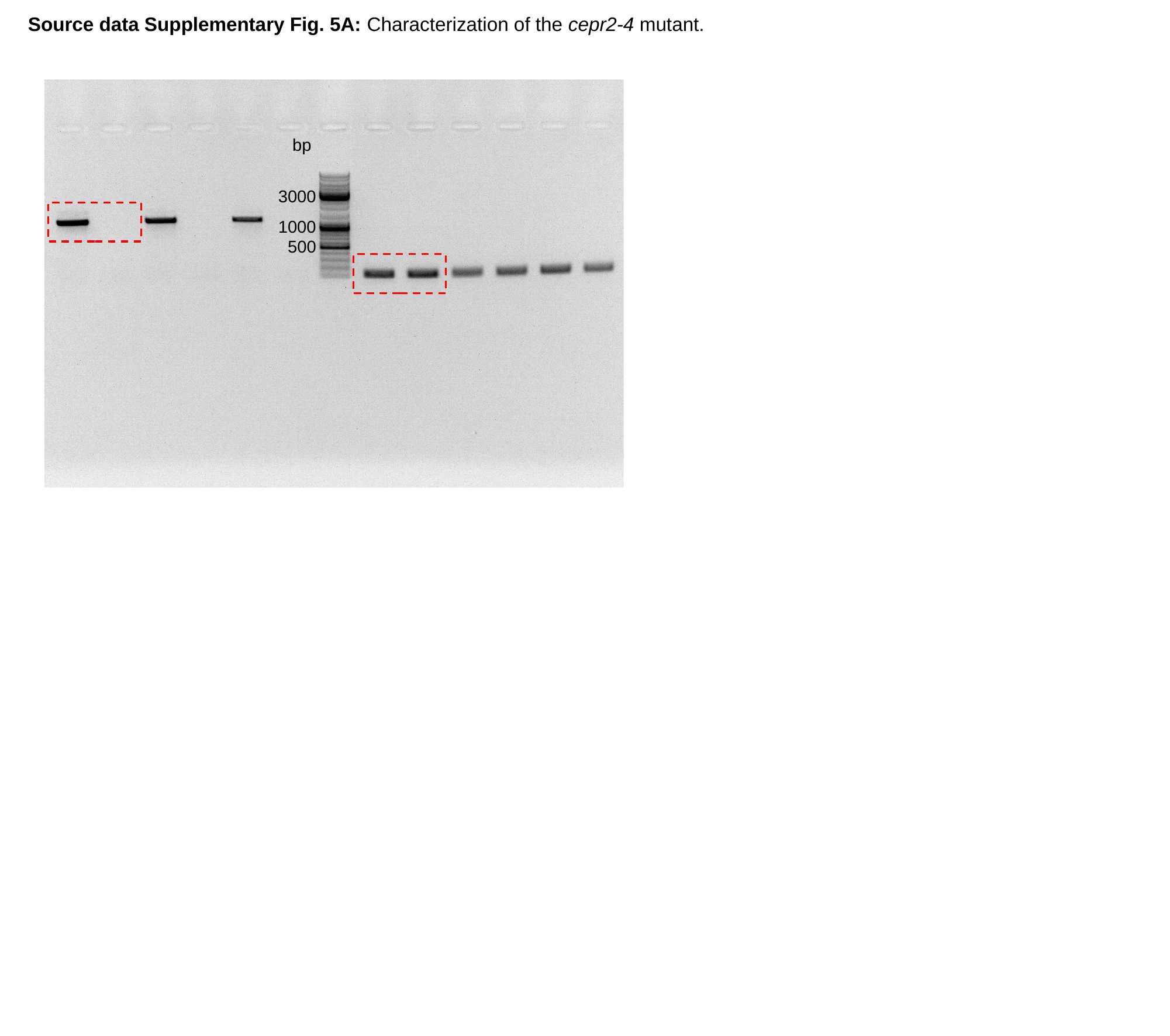

Source data Supplementary Fig. 5A: Characterization of the cepr2-4 mutant.
bp
3000
1000
500

## Slide 10
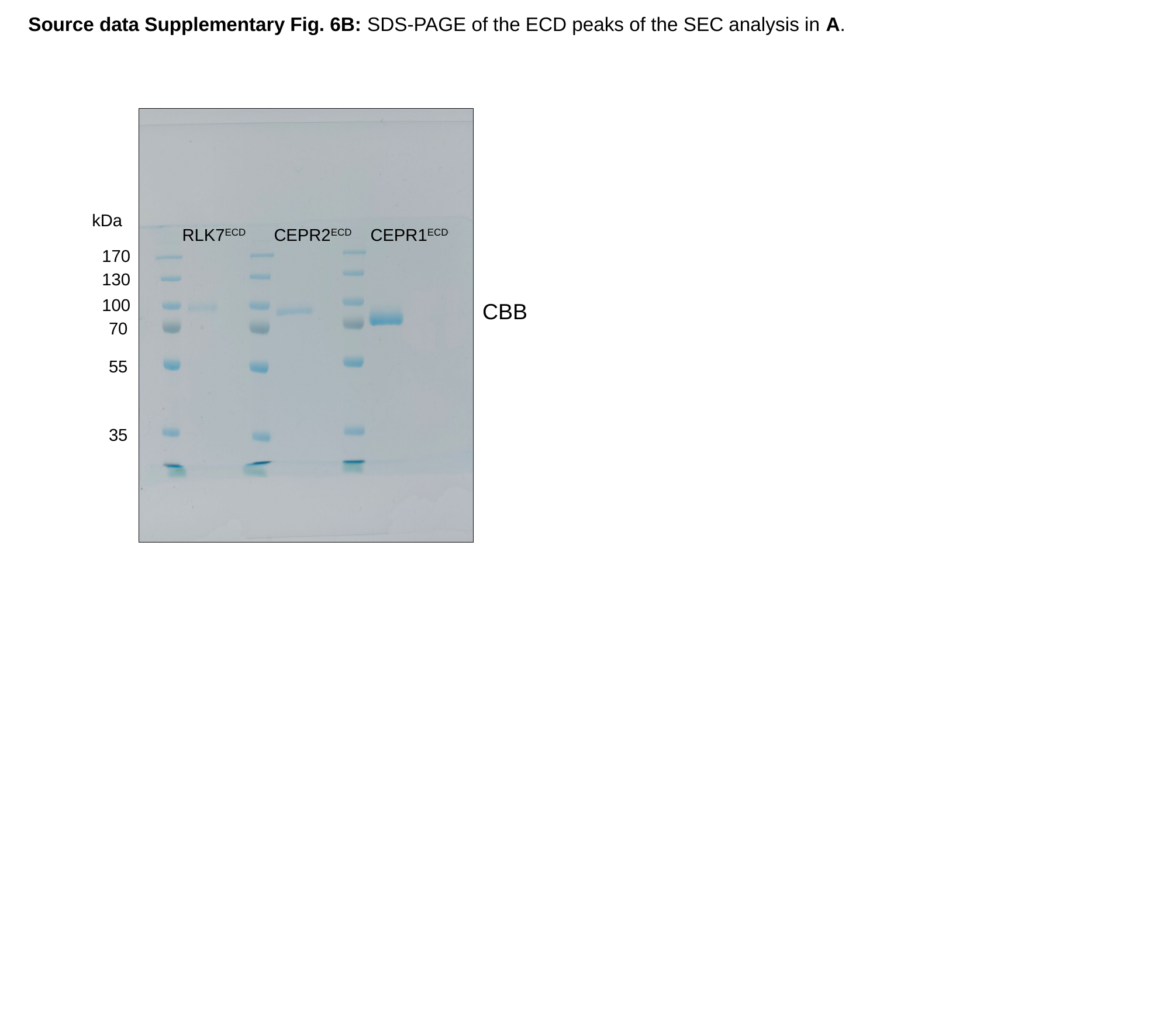

Source data Supplementary Fig. 6B: SDS-PAGE of the ECD peaks of the SEC analysis in A.
kDa
RLK7ECD
CEPR2ECD
CEPR1ECD
170
130
100
CBB
70
55
35

## Slide 11
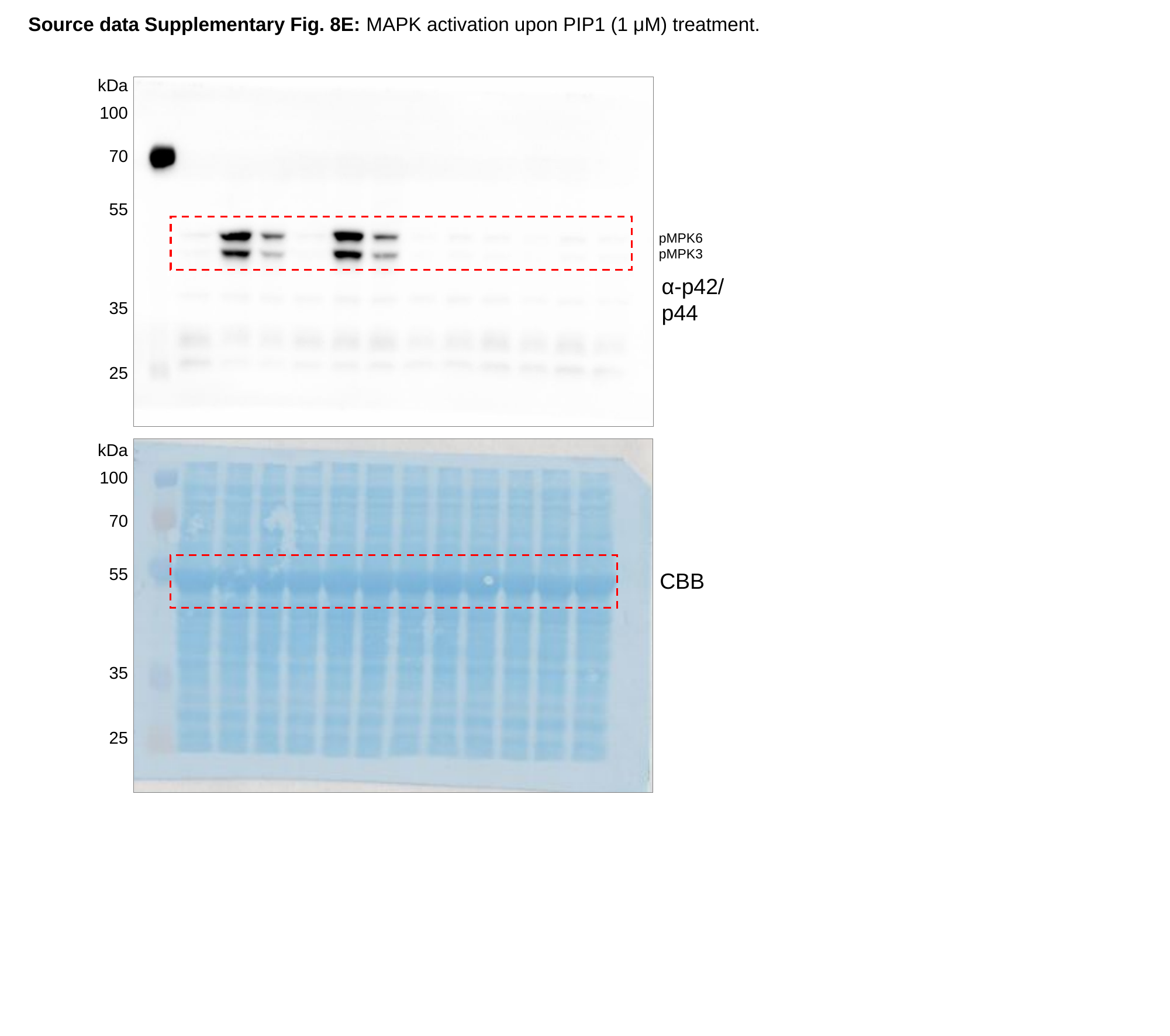

Source data Supplementary Fig. 8E: MAPK activation upon PIP1 (1 μM) treatment.
kDa
100
70
55
pMPK6
pMPK3
α-p42/
p44
35
25
kDa
100
70
55
CBB
35
25

## Slide 12
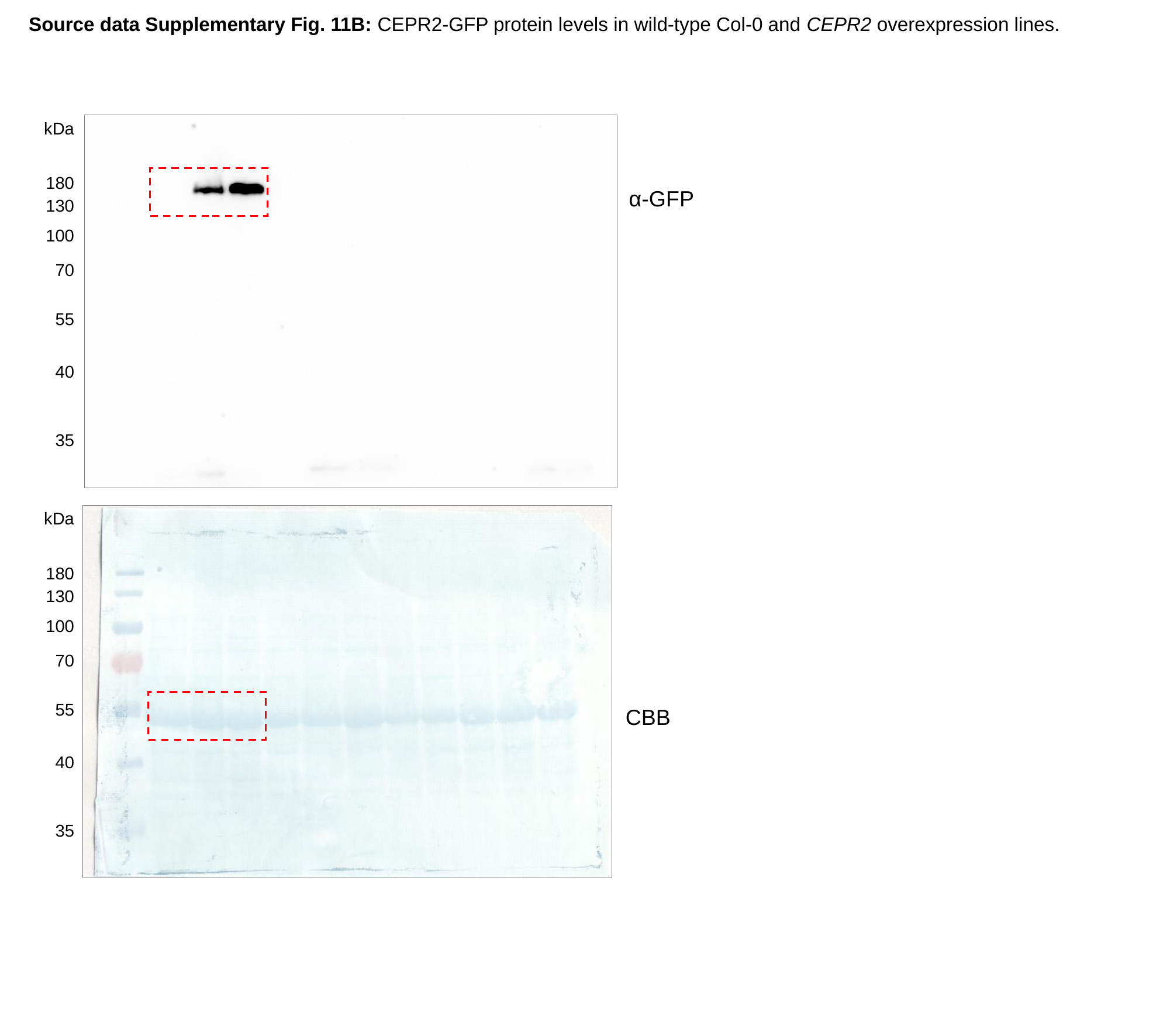

Source data Supplementary Fig. 11B: CEPR2-GFP protein levels in wild-type Col-0 and CEPR2 overexpression lines.
kDa
180
α-GFP
130
100
70
55
40
35
kDa
180
130
100
70
55
CBB
40
35

## Slide 13
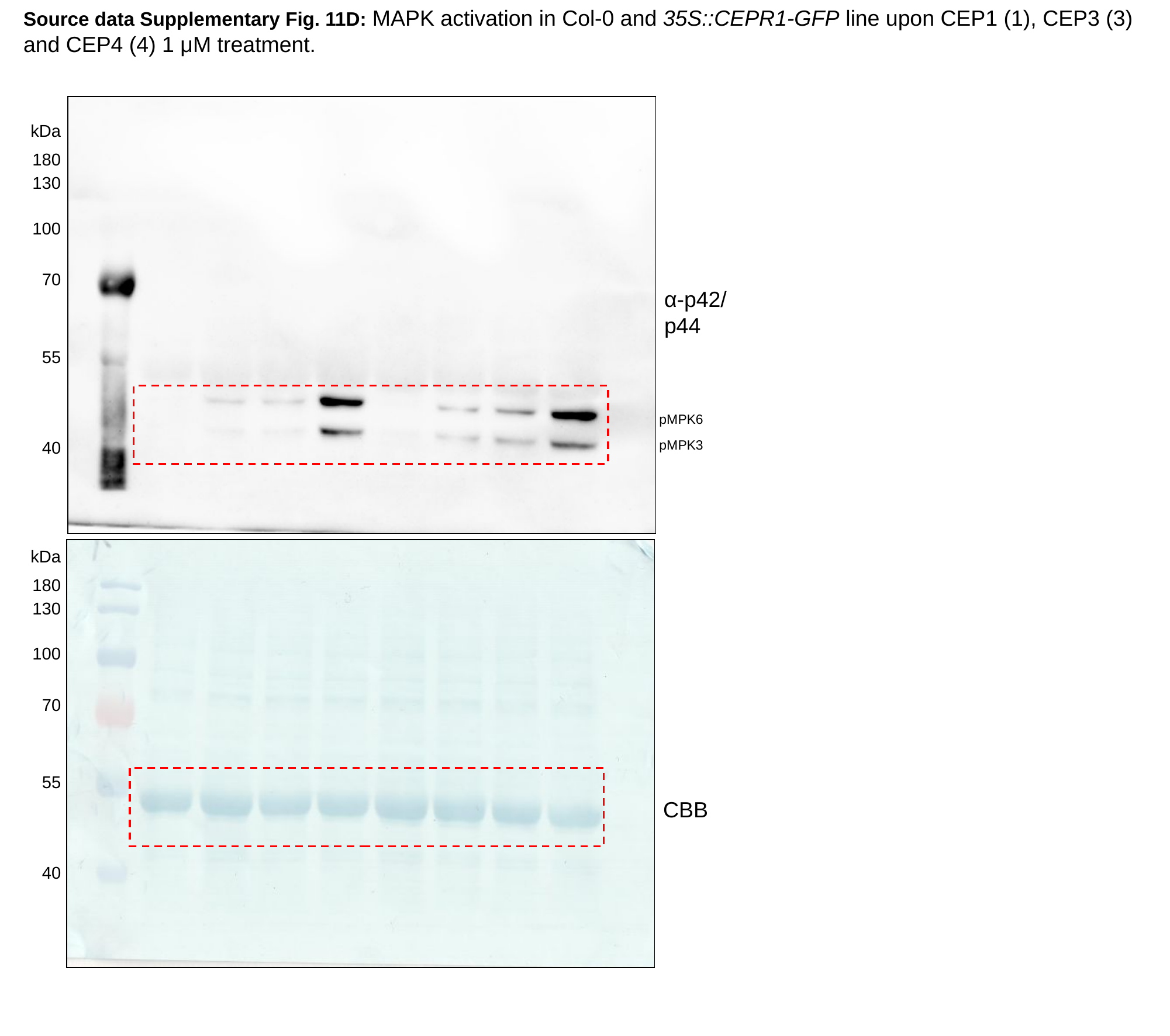

Source data Supplementary Fig. 11D: MAPK activation in Col-0 and 35S::CEPR1-GFP line upon CEP1 (1), CEP3 (3) and CEP4 (4) 1 μM treatment.
kDa
180
130
100
70
α-p42/
p44
55
pMPK6
pMPK3
40
kDa
180
130
100
70
55
CBB
40

## Slide 14
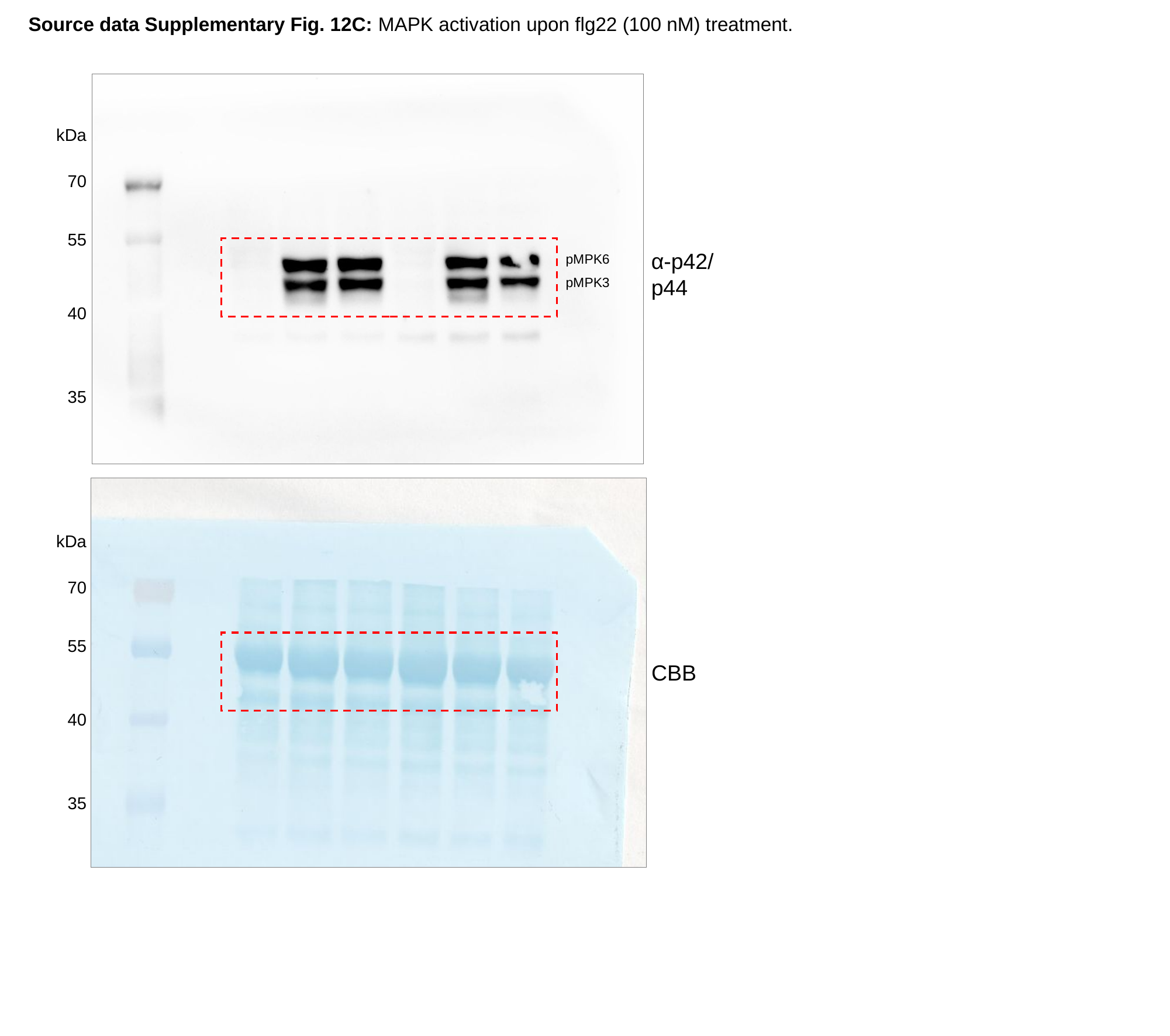

Source data Supplementary Fig. 12C: MAPK activation upon flg22 (100 nM) treatment.
kDa
70
55
α-p42/
p44
pMPK6
pMPK3
40
35
kDa
70
55
CBB
40
CBB
35

## Slide 15
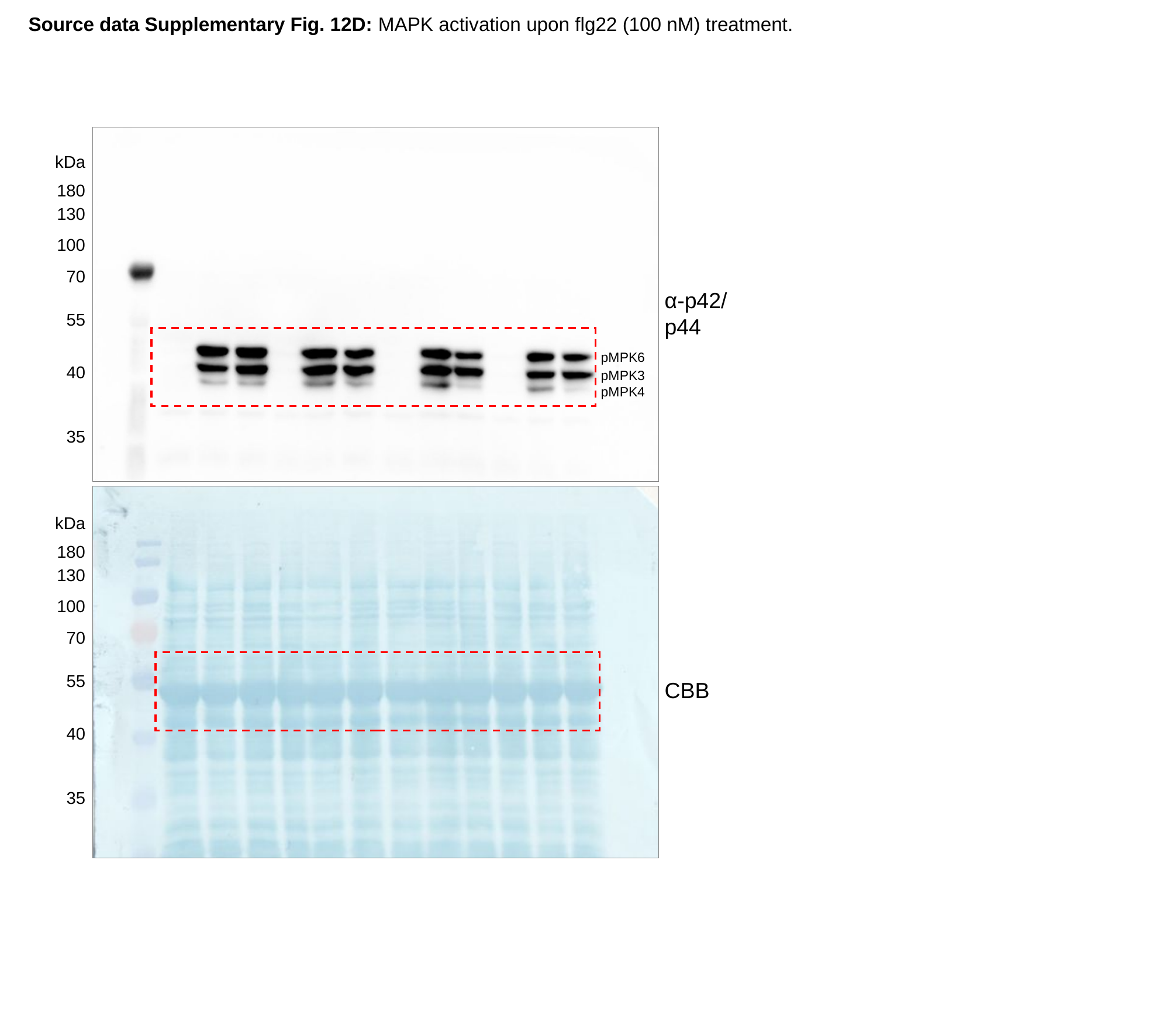

Source data Supplementary Fig. 12D: MAPK activation upon flg22 (100 nM) treatment.
kDa
180
130
100
70
α-p42/
p44
55
CBB
pMPK6
40
pMPK3
pMPK4
35
kDa
180
130
100
70
55
CBB
40
35

## Slide 16
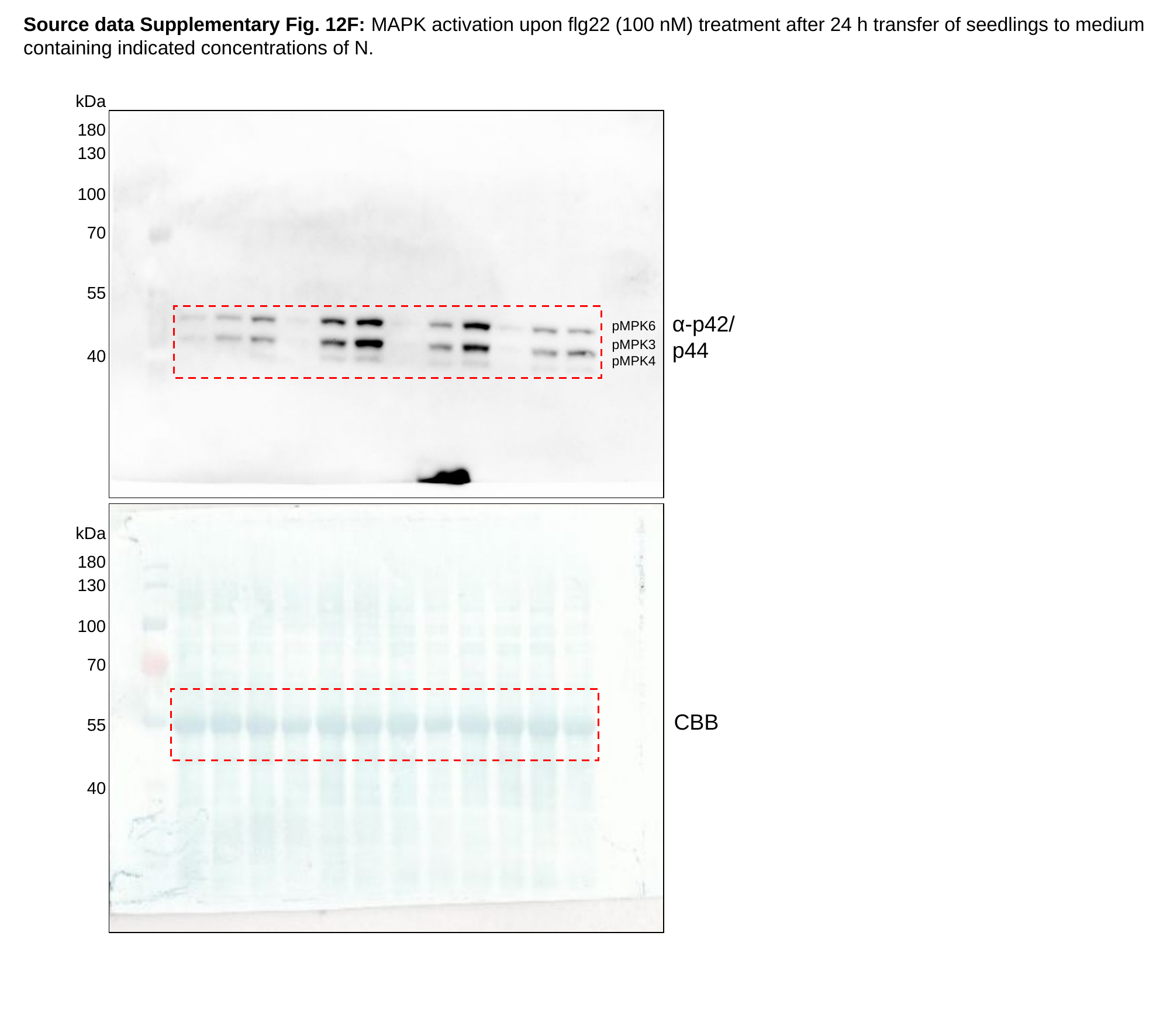

Source data Supplementary Fig. 12F: MAPK activation upon flg22 (100 nM) treatment after 24 h transfer of seedlings to medium containing indicated concentrations of N.
kDa
180
130
100
70
CBB
55
α-p42/
p44
pMPK6
pMPK3
40
pMPK4
kDa
180
130
100
70
CBB
55
40

## Slide 17
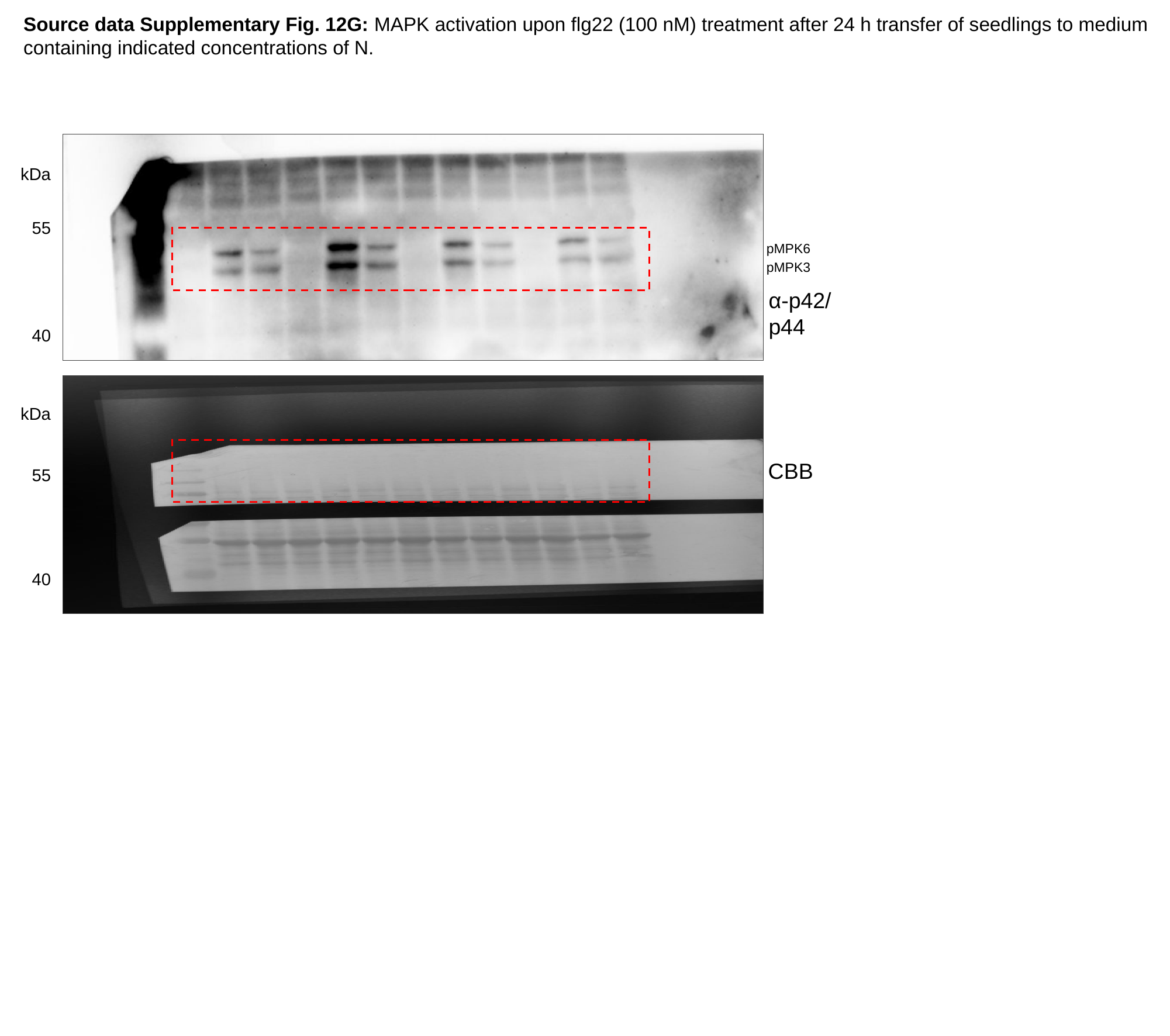

Source data Supplementary Fig. 12G: MAPK activation upon flg22 (100 nM) treatment after 24 h transfer of seedlings to medium containing indicated concentrations of N.
kDa
55
pMPK6
pMPK3
α-p42/
p44
40
kDa
CBB
55
40

## Slide 18
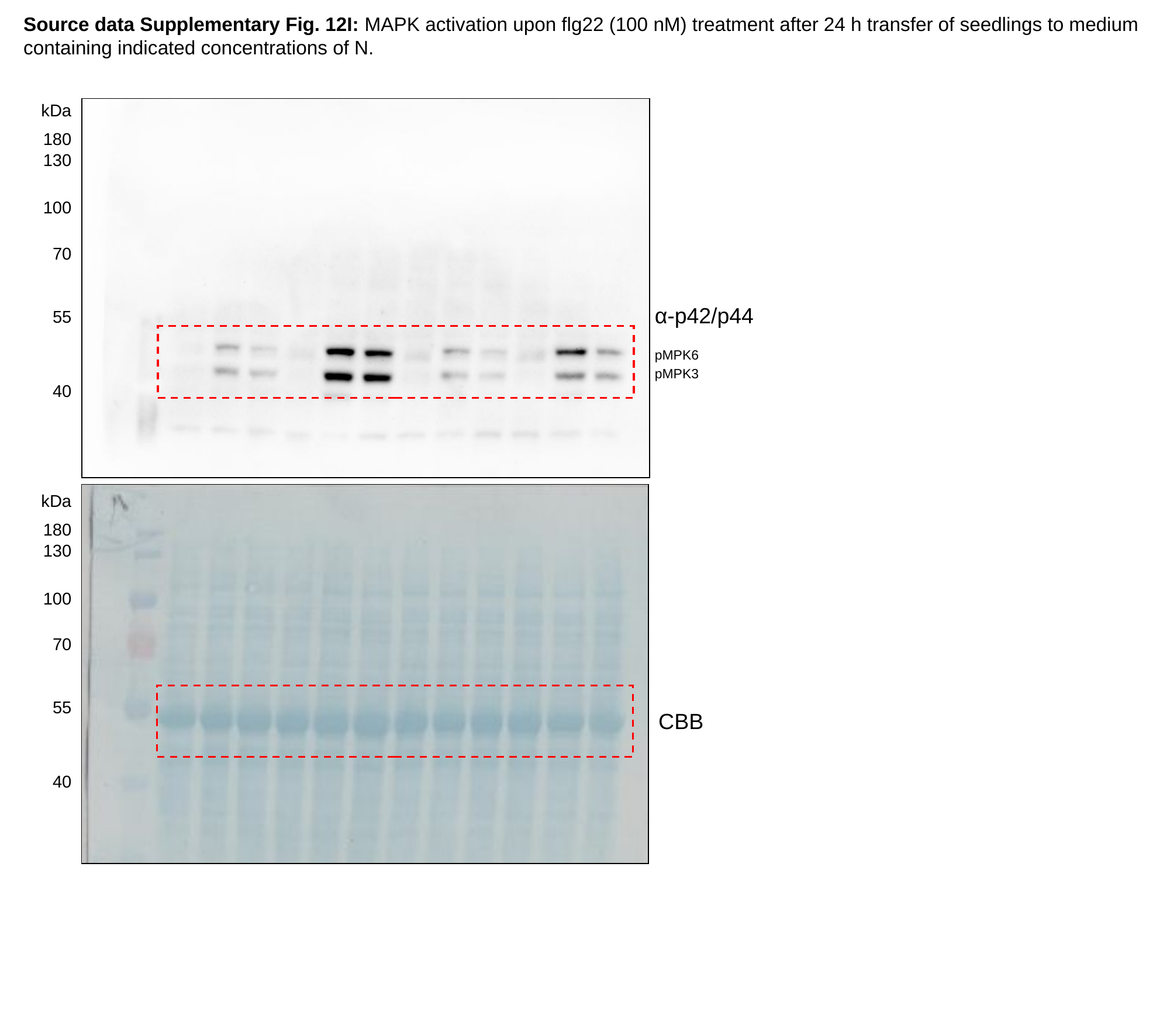

Source data Supplementary Fig. 12I: MAPK activation upon flg22 (100 nM) treatment after 24 h transfer of seedlings to medium containing indicated concentrations of N.
kDa
180
130
100
70
α-p42/p44
55
pMPK6
pMPK3
40
kDa
180
130
100
70
55
CBB
40
